# Alcohol consumption and all-cause and cause-specific mortality among US adults: prospective cohort study

**DOI:** 10.1186/s12916-023-02907-6

**Published:** 2023-06-07

**Authors:** Yalan Tian, Jiahui Liu, Yue Zhao, Nana Jiang, Xiao Liu, Gang Zhao, Xia Wang

**Affiliations:** 1 Department of Maternal and Child Health, School of Public Health, Cheeloo College of Medicine, Shandong University, 44 Wenhuaxi Road, Jinan, 250012 China; 2 Department of Cardiology, Shandong Provincial Hospital Affiliated to Shandong First Medical University, Jinan, China

**Keywords:** Alcohol, All-cause mortality, Cause-specific mortality, Cardiovascular disease, Cancer, Chronic lower respiratory tract diseases, Accidents (unintentional injuries), Alzheimer’s disease, Diabetes mellitus, Influenza and pneumonia, Nephritis, Nephrotic syndrome, Or nephrosis, Mortality

## Abstract

**Background:**

Previous studies have shown inconsistent findings regarding the association of light to moderate alcohol consumption with cause-specific mortality. Therefore, this study sought to examine the prospective association of alcohol consumption with all-cause and cause-specific mortality in the US population.

**Methods:**

This was a population-based cohort study of adults aged 18 years or older in the National Health Interview Survey (1997 to 2014) with linkage to the National Death Index records through December 31, 2019. Self-reported alcohol consumption was categorized into seven groups (lifetime abstainers; former infrequent or regular drinkers; and current infrequent, light, moderate, or heavy drinkers). The main outcome was all-cause and cause-specific mortality.

**Results:**

During an average follow-up of 12.65 years, among the 918,529 participants (mean age 46.1 years; 48.0% male), 141,512 adults died from all causes, 43,979 from cardiovascular disease (CVD), 33,222 from cancer, 8246 from chronic lower respiratory tract diseases, 5572 from accidents (unintentional injuries), 4776 from Alzheimer’s disease, 4845 from diabetes mellitus, 2815 from influenza and pneumonia, and 2692 from nephritis, nephrotic syndrome, or nephrosis. Compared with lifetime abstainers, current infrequent, light, or moderate drinkers were at a lower risk of mortality from all causes [infrequent—hazard ratio: 0.87; 95% confidence interval: 0.84 to 0.90; light: 0.77; 0.75 to 0.79; moderate 0.82; 0.80 to 0.85], CVD, chronic lower respiratory tract diseases, Alzheimer’s disease, and influenza and pneumonia. Also, light or moderate drinkers were associated with lower risk of mortality from diabetes mellitus and nephritis, nephrotic syndrome, or nephrosis. In contrast, heavy drinkers had a significantly higher risk of mortality from all causes, cancer, and accidents (unintentional injuries). Furthermore, binge drinking ≥ 1 day/week was associated with a higher risk of mortality from all causes (1.15; 1.09 to 1.22), cancer (1.22; 1.10 to 1.35), and accidents (unintentional injuries) (1.39; 1.11 to 1.74).

**Conclusions:**

Infrequent, light, and moderate alcohol consumption were inversely associated with mortality from all causes, CVD, chronic lower respiratory tract diseases, Alzheimer’s disease, and influenza and pneumonia. Light or moderate alcohol consumption might also have a beneficial effect on mortality from diabetes mellitus and nephritis, nephrotic syndrome, or nephrosis. However, heavy or binge had a higher risk of all-cause, cancer, and accidents (unintentional injuries) mortality.

**Supplementary Information:**

The online version contains supplementary material available at 10.1186/s12916-023-02907-6.

## Background

Alcohol is widely consumed in the United States and worldwide. The possible beneficial and detrimental effects of alcohol consumption, as investigated in many studies, have been hotly debated [1, 2]. Alcohol consumption has been linked to a range of health and social consequences [2]. Many studies have examined the association between alcohol consumption and all-cause, cardiovascular disease (CVD), and cancer mortality but with inconsistent findings [3–13]. In addition, most studies did not separately explore the specific types of other-causes mortality related to alcohol consumption, with the exception of all-cause, CVD, and cancer mortality [12–14]. Therefore, it is crucial to further confirm these previous research results as well as identify and clarify new relationships between alcohol consumption and disease/injury to inform policy efforts and prevention programs.

The harm caused by alcohol accounts for about one tenth of the total health impact of alcohol (9.9% and 12.6% in low- and high-income countries, respectively) [15]. The literature has reported that alcohol consumption is associated with a higher risk of injuries, mainly based on cross-sectional [16–19], case–crossover [20, 21], and case–control studies [22]. The magnitude of the effects in the alcohol and injury relationship may be impacted by the study design. Few prospective cohort studies have explored the risk of accidents (unintentional injuries) mortality due to drinking.

Some studies found that light or moderate alcohol consumption is associated with a lower risk of diabetes mellitus [23–25]. However, one meta-analysis showed that the reductions in risk were attenuated if former drinkers were excluded from the reference category [3]. Many studies may overestimate the degree of risk reduction for moderate drinkers as a result of comparing drinkers to a less healthy nondrinking reference category [26]. Furthermore, the association between alcohol consumption and the respiratory system remains controversial. A large population-based study in men found that alcohol use had a lower risk of death from overall respiratory disease and obstructive pulmonary disease (COPD) for all drinkers, but the study did not distinguish between moderate and occasional drinking [27]. Nevertheless, another study in individuals aged 12–41 years indicated that alcohol consumption had a U-shaped association with risk of new-onset asthma [28].

Few studies have examined the association between alcohol consumption and mortality due to Alzheimer’s disease. A Norwegian study examined the relationship between alcohol consumption and risk of dementia-related death, but the participants only comprised people aged between 60 and 80 years [29]. Some studies focused on the risk of alcohol-related dementia and cognitive decline, but they did not estimate the outcome of death due to Alzheimer’s disease [30, 31]. Additionally, several studies reported that alcohol consumption was related to chronic kidney disease [32, 33]. In contrast, one study found that alcohol consumption was not associated with an increased risk of renal dysfunction [34]. At present, no study has estimated the association between alcohol consumption and mortality from nephritis, nephrotic syndrome, or nephrosis.

The most recent national data related to alcohol consumption are available from the National Health Interview Survey (NHIS). Also, the National Center for Health Statistics (NCHS) has recently updated their information on the NHIS surveys linked to the National Death Index (NDI) data through December 31, 2019. Two previous studies on the NHIS [12, 13] only examined that the association of alcohol consumption with all-cause, CVD, and cancer mortality and current infrequent drinkers were also not separate from current light drinkers or lifetime abstainers. Therefore, we comprehensively estimated the recent association of alcohol consumption with all-cause and cause-specific mortality among a nationally representative sample of US adults.

## Methods

### Study population

The NHIS is a multi-purpose health survey of the civilian, noninstitutionalized, household population of the US continuously conducted by the NCHS and Centers for Disease Control and Prevention since 1957. A stratified, multistage probability sample design is used to represent the civilian noninstitutionalized US population. The NHIS data were collected through computer-assisted personal interviews administered by interviewers directed and trained by the US Census Bureau.

Information on basic health status was collected for all household members. One randomly sampled adult from each household was thoroughly interviewed about health and lifestyle behaviors, including health status, health behaviors, and healthcare utilization. Information on the study design, methodology, and weights is described in detail elsewhere [35].

This study used data from NHIS years that included alcohol consumption data from 1997 to 2014, with linkage to the NDI through December 31, 2019. All data obtained from the survey are publicly available on-line via the NCHS website [36]. We included participants aged 18 years and older at baseline with mortality follow-up information, including underlying cause of death.

### The assessment of study exposure

The questionnaires relating to alcohol use status and patterns of consumption were administered for all sample adults. Alcohol-related items in NHIS included the following: (1) In any 1 year, have you had at least 12 drinks of any type of alcoholic beverage? (2) In your entire life, have you had at least 12 drinks of any type of alcoholic beverage? (3) In the past year, how often did you drink any type of alcoholic beverage? (4) In the past year, on those days that you drank alcoholic beverages, on the average, how many drinks did you have? (5) In the past year, on how many days did you have 5 or more/4 or more drinks of any alcoholic beverage?

Based on the responses to questions about drinking alcoholic beverages, participants were categorized into seven alcohol consumption groups [37]: lifetime abstainer (< 12 drinks in one’s lifetime), former infrequent drinker (< 12 drinks in any previous year and none in the past year), former regular drinker (≥ 12 drinks in any previous year in one’s lifetime but none in the past year), current infrequent drinker (1–11 drinks in the past year), current light drinker (≥ 12 drinks in the past year but ≤ 3 drinks/week), current moderate drinker (> 3 drinks/week to ≤ 7 drinks/week for women and > 3 drinks/week to ≤ 14 drinks/week for men), and current heavy drinker (> 7 drinks/week for women and > 14 drinks/week for men). Data about binge drinking status was collected by using the responses to questions. The answers using unit as days per year were transferred into using unit as days per week (or month). One alcoholic drink-equivalent is defined as one that contains 14 g of pure alcohol (about 0.6 fluid ounces or 1.2 tablespoons), as is found in one 12 oz of beer (5% alcohol), one 5 oz glass of wine (12% alcohol), or one 1.5 oz shot of distilled spirits (40% alcohol) [37].

To avoid drinker misclassification errors, current infrequent drinkers were separate from current light drinkers or lifetime abstainers. No drinks in past year was classified as former drinkers in data years 1997 to 2000, which did not make a distinction between former infrequent and former regular drinkers. Former drinkers in data years 1997 to 2000 were classified as former regular drinkers. Thus, former infrequent drinkers in data years 1997 to 2000 were missing. Participants classified as “former unknown frequency drinker,” “current unknown level drinker,” or “drinking status unknown” were not included in this study.

### Mortality ascertainment

Linked mortality files provided mortality follow-up data from the month of the interview through December 31, 2019. Mortality information was obtained using a probabilistic match algorithm between the NHIS surveys and death certificate records in the NDI data [38]. All NHIS participants aged 18 years and older with sufficient identifying data were eligible for mortality follow-up. To reduce participant disclosure risk, the NCHS developed data-perturbation techniques. Information regarding participant vital status was not perturbed. Previous studies have confirmed the accuracy of information on mortality in the NDI records [39].

For the 1997–2014 NHIS, the cause-specific death categories included 9 groups selected from the 113 NCHS underlying cause-of-death recodes, whereas those of 2015–2018 NHIS only included 5 groups. Thus, data from the 2015–2018 NHIS were not included in this study. Underlying cause of death were coded using the 9th Revision International Statistical Classification of Diseases (ICD-9) through 1998, and the remainder for case definition was used according to the 10th Revision (ICD-10) from 1999 to the present. Considering changes between the 2 coding systems, causes of deaths occurring before 1999 were converted into comparable ICD-10–based underlying-cause-of-death groups [40]. The study outcomes were all-cause and cause-specific mortality (CVD, cancer, chronic lower respiratory tract diseases, accidents and injuries, Alzheimer’s disease, diabetes mellitus, influenza and pneumonia, and nephritis, nephrotic syndrome, or nephrosis). Please see the additional file for the ICD-10 codes (see Additional file 1: Table S1).

A total of 1,345,653 NHIS participants 18 years and older during 1997 to 2014 were included in the study. Among these, 427,124 were excluded due to participants with former unknown frequency, current unknown level, and drinking status unknown (*n* = 17,903), missing mortality follow-up information (*n* = 406,699), or missing data information on potential covariates (*n* = 2522), leading to a final analytical sample of 918,529 adults. Please see flow chart of the selection of study participants (see Additional file 1: Figure S1).

### Study covariates

A set of covariates available in all NHIS surveys was included as confounders to estimate alcohol consumption. Demographic characteristics included age, sex, race/ethnicity, education, and marital status. Lifestyle factors included body mass index, physical activity, and smoking status. Chronic health conditions included cancer, diabetes mellitus, heart disease, hypertension, stroke, asthma, emphysema, and chronic bronchitis.

Body mass index was calculated using self-reported height and weight as weight in kilograms divided by height in meters squared. Smoking status was estimated in the NHIS Sample Adult Files. Smoking was defined as three categories—never smoker, former smoker, and current smoker—using self-reported responses to the survey questions about smoking. Never smokers were defined as those who reported smoking fewer than 100 cigarettes in their entire life. Current smokers were defined as those who reported smoking at least 100 cigarettes in their life and were currently smoking every day or some days. Former smokers were defined as those who smoked more than 100 cigarettes during their lifetime but currently did not smoke at all.

In a separate part of the questionnaires, questions about physical activity were asked as part of the NHIS periodic adult prevention module. Participants were divided into three groups according to self-reported physical activity: high, moderate, and low levels of physical activity. Participants were defined as having a low level of physical activity if they never engaged in or were unable to engage in such activities for at least 10 min. Participants were defined as having a moderate level of physical activity if they engaged in moderate-intensity activity at least 5 times a week for at least 30 min per day, or both, meeting the criteria of physical activity guidelines [41]. Participants were defined as having a high level of physical activity if they performed vigorous-intensity activity 3 or more days per week for at least 20 min per day. As health status was determined in the family core interview, it may have been proxy-reported. Hypertension, heart disease, stroke, cancer, diabetes, asthma, emphysema, and chronic bronchitis were defined by using participants’ self-reported responses to physician diagnoses.

### Statistical analyses

All analyses were done to account for the complex, stratified, multistage cluster sampling design of the NHIS by using stratification, clustering, and sample weights in the NHIS data. The baseline characteristics of participants are obtained at the start of survey. To compare baseline characteristics among different groups, we used the *χ*^2^ test for categorical variables and analysis variance for continuous variables. Also, we calculated the distribution of alcohol consumption across different years from 1997 to 2014.

For primary analyses, we set lifetime abstainers as the reference group. We examined whether there was any association between alcohol consumption and mortality due to CVD, cancer, chronic lower respiratory disease (e.g., asthma, bronchitis, and emphysema), accidents (unintentional injuries), Alzheimer’s disease, diabetes mellitus, pneumonia and influenza, nephritis, nephrotic syndrome, or nephrosis, or any causes by using Cox proportional-hazards regression models to calculate the hazard ratio (HR) and 95% confidence intervals (CIs). With Cox regression, the influence of multiple predictors on the hazard, that is, risk of mortality, can be modeled. The model relies on two critical assumptions: the proportional hazards and the log-linearity of covariates. No violations to the proportional hazards assumption were found using Schoenfeld residual diagram. The data set contains no outliers. The above-mentioned associations were investigated by adjusting for the following covariates: age, sex, race, or ethnicity (model 1); model 1 plus education level, physical activity, body mass index, smoking status, hypertension, heart disease, stroke, cancer, diabetes, asthma, emphysema, or chronic bronchitis in a separate model (model 2). Model assumptions were checked for all the analyses.

Additionally, we performed the dose–response analysis to quantitatively estimate the association of current alcohol consumption (as a continuous variable) with all-cause and cause-specific mortality. A potential curve linear relation was assessed by using restricted cubic splines with four knots at the 5th, 35th, 65th, and 95th percentiles of the distribution [42]. The non-linear association of alcohol consumption with all-cause and cause-specific mortality using restricted cubic splines models [43]. Non-linearity was detected for using the likelihood ratio test to compare two models: one containing only a linear effect and the other also containing cubic spline terms [44].

Sensitivity analyses were conducted to confirm the findings of this study. First, we recalculated the estimates by excluding participants who died within the first 2 years (i.e., a 2-year lag). Second, we conducted the analyses by excluding individuals with physician-diagnosed diseases. Third, to examine the effects of missing data, a sensitivity analysis was conducted after multiple imputations for variables with missing values. The Markov chain Monte Carlo imputation assumes a normal distribution for the variables in the imputation model [45]. Reliable estimates can be obtained even when the distribution of variables is not normal. Fourth, to examine if there is abstainer biases, we recalculated HR estimates by using current infrequent drinkers as reference groups instead of abstainers as the reference group. Fifth, we estimated HRs for all-cause mortality according to drinking status in the individual NHIS survey, and then performed pooled analyses to obtain the summary estimates across survey years.

Furthermore, we performed stratified analyses to estimate whether the relationship between alcohol consumption and mortality varied by age (< 60 vs*.* ≥ 60), sex, race or ethnicity (white vs. non-white), and smoking status (never smoked vs. ever smoked). In addition, according to binge-drinking status, the participants were divided into 5 subgroups: lifetime abstainer, no binge drinking, binge drinking < 1 day/month, binge drinking < 1 day/week, and binge drinking ≥ 1 day/week. Cox proportional-hazards regression models were used to estimate the effect of binge drinking on all-cause and cause-specific mortality.

Stata software (version 15.1) was used for dose–response analysis. All other statistical analyses were performed using survey modules of SAS statistical software version 9.4 (SAS Institute, Inc., Cary, NC, USA). All *P* values were based on two-tailed testing, and *P* values less than 0.05 denoted statistical significance.

## Results

### Population characteristics

Among the 918,529 eligible adults in the NHIS (mean age 46.1 years, 52.0% women) in this study, 24.4% (*n* = 223,757) were lifetime abstainers at baseline in 1997, 6.8% (*n* = 62,185) were former infrequent drinkers, 8.6% (*n* = 78,769) were former regular drinkers, 13.3% (*n* = 122,441) were current infrequent drinkers, 28.8% (*n* = 264,067) were current light drinkers, 13.4% (*n* = 122,825) were current moderate drinkers, and 4.8% (*n* = 44,485) were current heavy drinkers. Compared with lifetime abstainers, current heavy drinkers were more likely to be young, men, non-Hispanic whites, current smokers, and unmarried, and have high levels of physical activity, greater than high school education, and normal weight; they were also more likely to have cancer but less likely to have diabetes, hypertension, and CVD at baseline. Table 1 presents the baseline characteristics of the participants according to alcohol consumption status. Also, this study showed that the distribution of alcohol consumption was consistent across different years from 1997 to 2014 (see Additional file 1: Table S2).

**Table 1:** Demographic characteristics according to baseline alcohol consumption status, NHIS 1997–2014

Characteristics	Lifetime abstainer	Former infrequent drinker	Former regular drinker	Current infrequent drinker	Current light drinker	Current moderate drinker	Current heavy drinker	*P* values
	**(*n* = 223,757)**	**(*n* = 62,185)**	**(*n* = 78,769)**	**(*n* = 122,441)**	**(*n* = 264,067)**	**(*n* = 122,825)**	**(*n* = 44,485)**	
**Age group (years)**								< 0.001
18–45	55,083 (50.9)	9925 (30.4)	13,265 (31.9)	32,482 (49.7)	85,727 (58.3)	38,280 (53.2)	13,803 (53.4)	
45–65	32,174 (27.0)	13,703 (40.9)	16,740 (38.3)	23,314 (35.5)	45,620 (31.6)	23,835 (33.8)	8927 (35.3)	
≥ 65	33,006 (22.2)	11,684 (28.7)	15,818 (29.8)	12,023 (14.8)	17,798 (10.1)	10,690 (13.0)	3295 (11.2)	
**Sex**								< 0.001
Men	33,979 (33.7)	13,418 (41.7)	23,057 (54.3)	21,381 (35.4)	69,199 (50.2)	51,502 (72.7)	13,892 (55.2)	
Women	86,284 (66.3)	21,894 (58.3)	22,766 (45.7)	46,438 (64.6)	79,946 (49.8)	21,303 (27.3)	12,133 (44.8)	
**Race/ethnicity**								< 0.001
Hispanic	29,962 (18.8)	5173 (11.4)	6214 (9.9)	11,220 (12.2)	23,211 (11.8)	9391 (9.7)	2964 (8.1)	
Non-Hispanic white	56,030 (54.9)	21,833 (69.5)	30,921 (74.8)	43,123 (71.0)	100,583 (74.1)	53,524 (79.2)	19,346 (80.3)	
Non-Hispanic black	23,408 (16.6)	6365 (13.9)	6840 (11.3)	9613 (11.2)	17,318 (9.1)	6998 (7.5)	2666 (8.0)	
Other	10,847 (9.7)	1941 (5.2)	1839 (4.0)	3858 (5.6)	8012 (5.0)	2886 (3.6)	1045 (3.6)	
**Education**								< 0.001
Less than high school	35,712 (26.2)	8576 (21.9)	12,431 (23.9)	10,210 (13.4)	16,868 (9.8)	8362 (10.1)	3935 (13.9)	
High school	35,983 (31.3)	11,291 (33.4)	14,329 (32.9)	19,883 (30.4)	36,007 (24.7)	17,676 (24.5)	7468 (29.6)	
More than high school	47,399 (42.5)	15,254 (44.7)	18,782 (43.2)	37,504 (56.2)	95,901 (65.5)	46,545 (65.5)	14,537 (56.4)	
**Marital status**								< 0.001
Married	52,840 (51.3)	16,582 (59.7)	21,746 (60.1)	33,882 (60.3)	72,400 (58.3)	33,513 (56.3)	9355 (45.7)	
Divorced/separated/widowed	34,703 (18.8)	12,505 (24.4)	16,448 (24.6)	18,137 (17.8)	31,771 (13.8)	15,415 (13.6)	6669 (17.5)	
Never married	32,380 (29.9)	6177 (15.9)	7557 (15.3)	15,684 (21.9)	44,718 (27.9)	23,748 (30.1)	9953 (36.8)	
**Smoking status**								< 0.001
Nonsmoker	98,271 (82.4)	17,906 (50.5)	17,220 (37.6)	38,867 (57.1)	82,805 (56.0)	31,389 (43.9)	7418 (28.6)	
Former smokers	10,959 (8.5)	10,070 (28.4)	18,372 (40.2)	14,565 (21.5)	33,444 (22.7)	20,360 (28.4)	6442 (25.4)	
Current smoking	10,883 (9.0)	7289 (21.2)	10,160 (22.2)	14,335 (21.4)	32,745 (21.3)	20,992 (27.8)	12,144 (46.0)	
**Physical activity**								< 0.001
Insufficient	82,467 (68.6)	23,390 (65.2)	29,580 (67.5)	38,660 (58.1)	70,048 (47.6)	30,635 (42.6)	12,506 (48.6)	
Active	11,784 (10.9)	4082 (12.0)	4612 (11.1)	9078 (14.2)	22,462 (15.9)	10,644 (15.7)	3340 (13.8)	
Highly active	21,521 (20.5)	7648 (22.8)	8638 (21.3)	17,435 (27.7)	51,012 (36.4)	28,865 (41.7)	9136 (37.6)	
**Body mass index category**								< 0.001
Normal weight	47,518 (41.0)	10,943 (31.0)	15,435 (33.2)	23,544 (34.7)	60,082 (40.0)	29,017 (39.0)	11,702 (44.8)	
Overweight	37,421 (30.4)	11,661 (33.1)	15,965 (35.2)	21,577 (32.1)	51,255 (34.7)	28,651 (39.8)	8892 (34.3)	
Obese	35,324 (28.6)	12,708 (35.9)	14,423 (31.6)	22,698 (33.2)	37,808 (25.3)	15,137 (21.1)	5431 (20.8)	
**Chronic diseases**								
CVD	17,544 (13.2)	8090 (21.9)	11,597 (24.2)	9351 (13.1)	14,489 (9.3)	7686 (10.2)	2847 (10.4)	< 0.001
Hypertension	37,963 (28.2)	15,494 (41.5)	18,954 (39.0)	20,384 (28.5)	32,488 (20.7)	17,650 (23.7)	7214 (26.6)	
Cancer	8497 (6.5)	4243 (11.7)	5783 (12.2)	5628 (8.1)	9434 (6.1)	5163 (7.0)	1951 (7.5)	< 0.001
Diabetes	13,056 (9.6)	6040 (16.1)	7247 (15.0)	6148 (8.7)	7046 (4.6)	2834 (3.7)	924 (3.3)	< 0.001
Chronic lower respiratory tract diseases	15,442 (12.9)	6652 (18.6)	8385 (17.9)	11,395 (16.7)	20,604 (13.7)	9307 (12.6)	4138 (15.4)	< 0.001

Values are numbers (percentages) unless stated otherwise

*NHIS* National Health Interview Survey

During 11,650,593 person-years of follow-up (an average follow-up of 12.65 years and a maximum follow-up of 22.75 years), 141,512 participants died from all causes, 43,979 from CVD, 33,222 from cancer, 8246 from chronic lower respiratory tract diseases, 5572 from accidents (unintentional injuries), 4776 from Alzheimer’s disease, 4845 from diabetes mellitus, 2815 from influenza and pneumonia, and 2692 from nephritis, nephrotic syndrome, or nephrosis.

### Alcohol consumption and all-cause and cause-specific mortality

Table 2 shows adjusted HRs for all-cause and cause-specific mortality according to alcohol consumption status at baseline. In the fully adjusted model (model 2), compared with lifetime abstainers, current infrequent, light, and moderate drinkers had a lower risk of all-cause mortality, with HRs of 0.87 (95% CI: 0.84 to 0.90), 0.77 (0.75 to 0.79), and 0.82 (0.80 to 0.85), respectively. Similarly, current infrequent, light, or moderate drinkers were associated with a reduced risk of mortality from CVD (infrequent: 0.86; 0.82 to 0.91; light: 0.76; 0.73 to 0.80; moderate: 0.78; 0.74 to 0.82), from chronic lower respiratory tract diseases (infrequent: 0.88; 0.77 to 0.99; light: 0.68; 0.60 to 0.76; moderate: 0.78; 0.68 to 0.90), from Alzheimer’s disease (infrequent: 0.76; 0.65 to 0.88; light: 0.68; 0.59 to 0.78; moderate: 0.83; 0.69 to 0.99), and from influenza and pneumonia (infrequent: 0.69; 0.57 to 0.83; light: 0.63; 0.52 to 0.75; moderate: 0.58; 0.46 to 0.73). This study also found that light or moderate drinkers had a reduced risk of mortality from diabetes mellitus (light: 0.72; 0.61 to 0.84; moderate 0.73; 0.60 to 0.88) and nephritis, nephrotic syndrome, or nephrosis (light: 0.66; 0.54 to 0.81; moderate 0.62; 0.48 to 0.79).

**Table 2:** All-cause and cause-specific mortality (lifetime abstainer as reference group) according to alcohol consumption status among NHIS participants in 1997 to 2014

	**Lifetime abstainer**	**Former infrequent drinker**	**Former regular drinker**	**Current infrequent drinker**	**Current light drinker**	**Current moderate drinker**	**Current heavy drinker**
	(*n* = 223,757)	(*n* = 62,185)	(*n* = 78,769)	(*n* = 122,441)	(*n* = 264,067)	(*n* = 122,825)	(*n* = 44,485)
**All-cause**							
Deaths	40,479	12,727	23,551	17,031	26,053	14,977	6694
Person-years	2,798,158	635,930	1,029,426	1,572,351	3,444,244	1,551,492	572,793
Model 1	1 (ref)	1.15 (1.11–1.19)	1.28 (1.24–1.31)	0.90 (0.87–0.93)	0.73 (0.71–0.75)	0.80 (0.78–0.83)	1.27 (1.21–1.33)
Model 2	1 (ref)	1.00 (0.97–1.03)	1.08 (1.05–1.11)	0.87 (0.84–0.90)	0.77 (0.75–0.79)	0.82 (0.80–0.85)	1.07 (1.03–1.12)
2-yr lag model	1 (ref)	0.99 (0.96–1.03)	1.07 (1.04–1.10)	0.87 (0.84–0.90)	0.78 (0.75–0.80)	0.84 (0.81–0.87)	1.08 (1.03–1.13)
**CVD**							
Deaths	13,562	4041	7659	5216	7585	4242	1674
Person-years	2,798,158	635,930	1,029,426	1,572,351	3,444,244	1,551,492	572,793
Model 1	1 (ref)	1.08 (1.02–1.15)	1.23 (1.17–1.29)	0.87 (0.82–0.91)	0.67 (0.64–0.71)	0.69 (0.65–0.73)	0.99 (0.91–1.08)
Model 2	1 (ref)	0.97 (0.92–1.03)	1.08 (1.03–1.13)	0.86 (0.82–0.91)	0.76 (0.73–0.80)	0.78 (0.74–0.82)	0.94 (0.86–1.01)
2-yr lag model	1 (ref)	0.98 (0.92–1.04)	1.07 (1.02–1.13)	0.88 (0.83–0.92)	0.77 (0.73–0.81)	0.79 (0.75–0.84)	0.96 (0.89–1.05)
**Cancer**							
Deaths	8169	2792	5254	4156	6851	4072	1928
Person-years	2,798,158	635,930	1,029,426	1,572,351	3,444,244	1,551,492	572,793
Model 1	1 (ref)	1.34 (1.25–1.44)	1.51 (1.42–1.60)	1.13 (1.06–1.21)	0.98 (0.93–1.04)	1.13 (1.06–1.21)	1.89 (1.73–2.06)
Model 2	1 (ref)	1.05 (0.98–1.12)	1.15 (1.08–1.21)	0.95 (0.89–1.01)	0.86 (0.81–0.91)	0.93 (0.87–1.00)	1.24 (1.14–1.34)
2-yr lag model	1 (ref)	1.06 (0.99–1.14)	1.14 (1.07–1.22)	0.96 (0.90–1.03)	0.88 (0.83–0.94)	0.96 (0.90–1.03)	1.28 (1.18–1.40)
**Chronic lower respiratory tract diseases**							
Deaths	2030	865	1675	1058	1323	840	455
Person-years	2,798,158	635,930	1,029,426	1,572,351	3,444,244	1,551,492	572,793
Model 1	1 (ref)	1.65 (1.45–1.87)	2.00 (1.80–2.22)	1.15 (1.02–1.29)	0.77 (0.69–0.87)	0.94 (0.83–1.07)	1.78 (1.53–2.07)
Model 2	1 (ref)	0.99 (0.88–1.12)	1.09 (0.98–1.21)	0.88 (0.77–0.99)	0.68 (0.60–0.76)	0.78 (0.68–0.90)	0.95 (0.81–1.10)
2-yr lag model	1 (ref)	0.98 (0.85–1.12)	1.08 (0.96–1.21)	0.87 (0.76–0.99)	0.67 (0.59–0.77)	0.78 (0.67–0.89)	0.96 (0.82–1.12)
**Accidents and injuries**							
Deaths	1331	425	725	685	1312	697	397
Person-years	2,798,158	635,930	1,029,426	1,572,351	3,444,244	1,551,492	572,793
Model 1	1 (ref)	1.08 (0.89–1.31)	1.15 (0.99–1.34)	0.79 (0.67–0.93)	0.86 (0.74–0.99)	0.85 (0.73–1.00)	1.70 (1.39–2.08)
Model 2	1 (ref)	1.08 (0.90–1.29)	1.04 (0.90–1.20)	0.85 (0.72–0.99)	0.96 (0.83–1.11)	0.90 (0.77–1.06)	1.48 (1.22–1.80)
2-yr lag model	1 (ref)	0.96 (0.79–1.16)	0.99 (0.85–1.16)	0.78 (0.66–0.92)	0.93 (0.80–1.08)	0.87 (0.74–1.02)	1.34 (1.09–1.65)
**Alzheimer’s disease**							
Deaths	1730	448	715	517	782	442	142
Person-years	2,798,158	635,930	1,029,426	1,572,351	3,444,244	1,551,492	572,793
Model 1	1 (ref)	1.04 (0.89–1.21)	0.96 (0.84–1.09)	0.74 (0.64–0.86)	0.71 (0.62–0.82)	0.88 (0.75–1.04)	0.73 (0.49–1.09)
Model 2	1 (ref)	1.05 (0.90–1.23)	0.95 (0.83–1.09)	0.76 (0.65–0.88)	0.68 (0.59–0.78)	0.83 (0.69–0.99)	0.84 (0.70–1.00)
2-yr lag model	1 (ref)	1.01 (0.86–1.19)	0.92 (0.80–1.05)	0.73 (0.63–0.85)	0.69 (0.60–0.79)	0.84 (0.70–1.00)	0.85 (0.68–1.08)
**Diabetes mellitus**							
Deaths	1574	504	927	591	747	364	138
Person-years	2,798,158	635,930	1,029,426	1,572,351	3,444,244	1,551,492	572,793
Model 1	1 (ref)	1.16 (0.98–1.38)	1.39 (1.22–1.58)	0.86 (0.75–1.00)	0.46 (0.39–0.53)	0.41 (0.34–0.50)	0.59 (0.43–0.82)
Model 2	1 (ref)	0.92 (0.79–1.07)	1.17 (1.02–1.33)	0.92 (0.79–1.08)	0.72 (0.61–0.84)	0.73 (0.60–0.88)	0.93 (0.66–1.29)
2-yr lag model	1 (ref)	0.90 (0.75–1.08)	1.16 (1.00–1.33)	0.93 (0.79–1.08)	0.70 (0.59–0.82)	0.72 (0.59–0.88)	0.90 (0.64–1.27)
**Influenza and pneumonia**							
Deaths	952	253	477	320	465	239	109
Person-years	2,798,158	635,930	1,029,426	1,572,351	3,444,244	1,551,492	572,793
Model 1	1 (ref)	0.77 (0.61–0.96)	1.11 (0.94–1.30)	0.67 (0.55–0.82)	0.57 (0.48–0.67)	0.53 (0.43–0.67)	0.88 (0.62–1.25)
Model 2	1 (ref)	0.73 (0.59–0.91)	0.98 (0.83–1.15)	0.69 (0.57–0.83)	0.63 (0.52–0.75)	0.58 (0.46–0.73)	0.80 (0.57–1.13)
2-yr lag model	1 (ref)	0.70 (0.55–0.88)	1.00 (0.84–1.19)	0.69 (0.56–0.84)	0.62 (0.52–0.74)	0.58 (0.46–0.74)	0.81 (0.56–1.16)
**Nephritis, nephrotic syndrome, or nephrosis**							
Deaths	895	284	474	330	428	184	97
Person-years	2,798,158	635,930	1,029,426	1,572,351	3,444,244	1,551,492	572,793
Model 1	1 (ref)	1.12 (0.92–1.37)	1.12 (0.94–1.34)	0.99 (0.81–1.22)	0.54 (0.44–0.65)	0.48 (0.37–0.62)	0.87 (0.61–1.23)
Model 2	1 (ref)	0.98 (0.81–1.17)	0.95 (0.80–1.12)	1.02 (0.84–1.23)	0.66 (0.54–0.81)	0.62 (0.48–0.79)	0.98 (0.71–1.36)
2-yr lag model	1 (ref)	1.01 (0.82–1.23)	0.97 (0.81–1.17)	1.07 (0.87–1.32)	0.69 (0.56–0.84)	0.65 (0.50–0.84)	1.04 (0.73–1.47)

Model 1: Adjusted for sex, age, and race/ethnicity

Model 2: Model 1 + Adjusted for education, marital status, body mass index, smoking status, physical activity, chronic conditions (heart disease, stroke, cancer, diabetes, hypertension, asthma, emphysema, and chronic bronchitis)

2-yr lag model: Model 2 + Lagged analyses exclude individuals who died within the first 2 years after administration of the respective NHIS

*NHIS* National Health Interview Survey

However, heavy drinkers had a higher risk of mortality from all causes (1.07; 1.03 to 1.12) and accidents (unintentional injuries) (1.48; 1.22 to 1.80). Although light drinkers were associated with a reduced risk of mortality from cancer (0.86; 0.81 to 0.91), heavy drinkers had an obviously higher risk of mortality from cancer (1.24; 1.14 to 1.34).

### Dose–response analysis

**Fig. 1:**
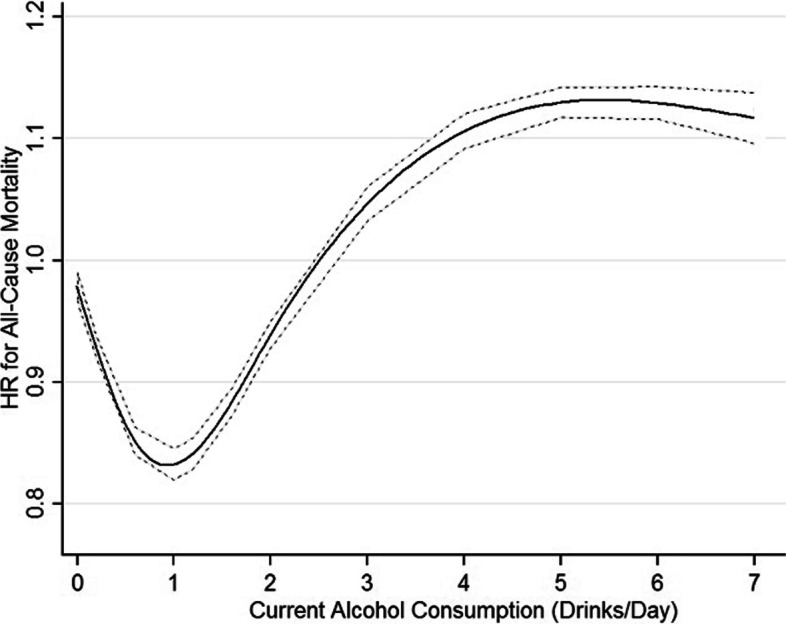
Dose–response relationship between alcohol consumption and risk of mortality from all causes. A nonlinear relationship of current alcohol consumption (as a continuous variable) with all-cause mortality (*p* < 0.05 for the nonlinear test), using maximally adjusted estimates (adjusted for sex, age, race/ethnicity, education, marital status, body mass index, physical activity, smoking, and physician-diagnosed diseases (heart disease, stroke, cancer, diabetes, hypertension, asthma, emphysema, and chronic bronchitis). That indicates that alcohol consumption had a J-shaped association with risk of all-cause mortality. HR, hazard ratio

This study performed a dose–response analysis between current alcohol consumption (as a continuous variable) and all-cause and cause-specific mortality. Figure 1 and Additional file 1 (Figures S2–S9) illustrate that a nonlinear association of current alcohol consumption with all-cause and cause-specific mortality (all *p* < 0.05 for the nonlinear test). Figure 1 indicates that alcohol consumption had a J-shaped association with risk of all-cause mortality. These findings were corresponding to those when current alcohol consumption was regarded as a category variable (lifetime abstainers, and current infrequent, light, moderate, and heavy drinkers) in Table 2.

### Sensitivity analyses

We performed the 2-year lag analysis to remove the effect of early deaths on the results. Further excluding early deaths in the 2-year lag analysis had little effect on the observed summary estimates (Table 2). Also, we analyzed again the dataset after multiple imputations for all variables with missing values, and similar results were obtained (see Additional file 1: Table S3).

In addition, we calculate HR estimates by using current infrequent drinkers as reference groups (see Additional file 1: Table S4). We still observed favorable effects of current light or moderate drinking on mortality from all causes, CVD, diabetes mellitus, and nephritis, nephrotic syndrome, or nephrosis. The harmful effects of heavy drinking on mortality from all causes, cancer, and accidents (unintentional injuries) seem to be more evident.

Furthermore, we estimated HRs for all-cause mortality according to drinking status in the individual NHIS survey, and then performed pooled analyses to obtain the summary estimates across survey years (see Additional file 1: Table S5). The results were in agreement with those in primary analyses. To further confirm the robustness of our findings, we recalculated the risk estimates by excluding participants with physician-diagnosed diseases (see Additional file 1: Table S6). The positive associations between former regular drinking and all-cause, CVD, cancer, and diabetes mellitus mortality seen previously were not observed. The negative associations between current infrequent drinking and mortality from lower respiratory tract diseases, Alzheimer’s disease, and influenza and pneumonia and between current moderate drinking and mortality from diabetes mellitus and nephritis, nephrotic syndrome, or nephrosis also disappeared. In general, the impact of excluding participants with physician-diagnosed diseases on the estimates of other associations was small.

### Stratification analysis

Stratified analyses by sex, age, race, and smoke status were conducted to further verify the findings of this study (see Additional file 1: Table S7). The results showed that the associations between alcohol consumption and mortality risk varied with sex, age, race, and smoke status. A protective effect of light or moderate drinking on mortality was more pronounced in women than in men. In contrast, current heavy drinkers had a higher risk of all-cause and cancer mortality in men but not in women.

Favorable effects of current infrequent, light, or moderate drinking on mortality from Alzheimer’s disease were observed in participants older than 60 but not in those younger than 60. Beneficial effects of moderate drinking on mortality from all causes, CVD, cancer, chronic lower respiratory tract diseases, Alzheimer’s disease, and diabetes mellitus were found in non-Hispanic white subjects but not in other ethnic groups.

Furthermore, the protective effect of light or moderate drinking on mortality, particularly mortality from influenza and pneumonia, was more obvious in never smokers than in those who ever smoked. Meanwhile, the detrimental effect of heavy drinking on mortality from all causes, cancer, and accidents (unintentional injuries) was also more pronounced in those who had ever smoked than in never smokers.

### Binge drinking and all-cause and cause-specific mortality

Compared with lifetime abstainers, participants with binge drinking ≥ 1 day/week were associated with an increased risk of mortality from all causes (1.15; 1.09 to 1.22), cancer (1.22; 1.10 to 1.35), and accidents (unintentional injuries) (1.39; 1.11 to 1.74) in the multivariable adjustment model but were not associated with the risk of mortality from CVD, chronic lower respiratory tract diseases, Alzheimer’s disease, diabetes mellitus, influenza and pneumonia, and nephritis, nephrotic syndrome, or nephrosis. The associations were not significant changed for all-cause and cause-specific mortality in the 2-year lag analysis (Table 3).

**Table 3:** All-cause and cause-specific mortality according to binge drinking status among NHIS participants in 1997 to 2014

	**Lifetime abstainer**	**No binge drinking**	**Binge drinking < 1 day/month**	**Binge drinking < 1 day/week**	**Binge drinking ≥ 1 day/week**
**All-cause**					
Model 1	1 (ref)	0.80 (0.78–0.82)	0.87 (0.83–0.91)	0.98 (0.91–1.06)	1.52 (1.44–1.61)
Model 2	1 (ref)	0.84 (0.81–0.86)	0.83 (0.79–0.87)	0.85 (0.78–0.92)	1.15 (1.09–1.22)
2-yr lag model	1 (ref)	0.84 (0.82–0.87)	0.85 (0.81–0.89)	0.87 (0.80–0.94)	1.17 (1.10–1.24)
**CVD**					
Model 1	1 (ref)	0.75 (0.71–0.78)	0.87 (0.80–0.95)	1.03 (0.90–1.18)	1.25 (1.13–1.39)
Model 2	1 (ref)	0.85 (0.81–0.89)	0.91 (0.84–1.00)	0.98 (0.85–1.12)	1.05 (0.95–1.17)
2-yr lag model	1 (ref)	0.86 (0.82–0.90)	0.92 (0.84–1.01)	1.00 (0.87–1.15)	1.10 (0.98–1.23)
**Cancer**					
Model 1	1 (ref)	1.09 (1.03–1.15)	1.10 (1.01–1.20)	1.12 (0.97–1.29)	1.91 (1.73–2.12)
Model 2	1 (ref)	0.94 (0.88–1.00)	0.85 (0.78–0.93)	0.80 (0.69–0.93)	1.22 (1.10–1.35)
2-yr lag model	1 (ref)	0.97 (0.91–1.04)	0.90 (0.82–0.99)	0.85 (0.73–0.98)	1.24 (1.12–1.39)
**Chronic lower respiratory tract diseases**					
Model 1	1 (ref)	0.95 (0.84–1.06)	1.17 (0.97–1.41)	1.14 (0.84–1.57)	2.51 (2.04–3.10)
Model 2	1 (ref)	0.79 (0.69–0.89)	0.82 (0.68–0.99)	0.67 (0.49–0.92)	1.19 (0.96–1.47)
2-yr lag model	1 (ref)	0.77 (0.68–0.88)	0.81 (0.66–0.99)	0.71 (0.52–0.98)	1.21 (0.97–1.51)
**Accidents/injuries**					
Model 1	1 (ref)	0.87 (0.76–1.00)	1.06 (0.89–1.27)	1.25 (0.95–1.64)	1.94 (1.56–2.40)
Model 2	1 (ref)	0.94 (0.81–1.10)	1.05 (0.86–1.27)	1.05 (0.80–1.39)	1.39 (1.11–1.74)
2-yr lag model	1 (ref)	0.89 (0.76–1.04)	1.03 (0.84–1.26)	1.00 (0.74–1.35)	1.36 (1.09–1.69)
**Alzheimer’s disease**					
Model 1	1 (ref)	0.72 (0.64–0.82)	0.38 (0.26–0.55)	0.44 (0.21–0.96)	0.83 (0.47–1.45)
Model 2	1 (ref)	0.71 (0.62–0.81)	0.38 (0.26–0.56)	0.45 (0.21–0.97)	0.80 (0.45–1.45)
2-yr lag model	1 (ref)	0.71 (0.62–0.81)	0.38 (0.26–0.57)	0.46 (0.21–1.00)	0.83 (0.46–1.49)
**Diabetes mellitus**					
Model 1	1 (ref)	0.56 (0.49–0.64)	0.59 (0.45–0.77)	0.46 (0.29–0.74)	0.91 (0.68–1.23)
Model 2	1 (ref)	0.89 (0.77–1.03)	0.94 (0.71–1.25)	0.66 (0.41–1.06)	1.19 (0.87–1.63)
2-yr lag model	1 (ref)	0.88 (0.76–1.02)	0.95 (0.71–1.27)	0.67 (0.41–1.09)	1.19 (0.86–1.64)
**Influenza and pneumonia**					
Model 1	1 (ref)	0.66 (0.56–0.78)	0.49 (0.32–0.75)	0.49 (0.27–0.89)	0.75 (0.48–1.17)
Model 2	1 (ref)	0.72 (0.60–0.85)	0.49 (0.32–0.75)	0.45 (0.25–0.82)	0.61 (0.38–0.95)
2-yr lag model	1 (ref)	0.72 (0.60–0.85)	0.52 (0.34–0.80)	0.46 (0.25–0.85)	0.61 (0.38–1.00)
**Nephritis, nephrotic syndrome, or nephrosis**					
Model 1	1 (ref)	0.62 (0.52–0.74)	0.50 (0.36–0.70)	0.77 (0.40–1.47)	1.02 (0.69–1.51)
Model 2	1 (ref)	0.81 (0.67–0.99)	0.63 (0.45–0.89)	0.89 (0.46–1.71)	1.04 (0.70–1.56)
2-yr lag model	1 (ref)	0.85 (0.70–1.04)	0.69 (0.49–0.99)	1.00 (0.52–1.91)	1.14 (0.75–1.73)

Model 1: Adjusted for sex, age, and race/ethnicity

Model 2: Model 1 + Adjusted for education, marital status, body mass index, smoking status, physical activity, chronic conditions (heart disease, stroke, cancer, diabetes, hypertension, asthma, emphysema, and chronic bronchitis)

2-yr lag model: Model 2 + Lagged analyses exclude individuals who died within the first 2 years after administration of the respective NHIS

## Discussion

In this nationally representative cohort study, we found that infrequent, light, or moderate alcohol consumption were associated with a lower risk of mortality from all causes, CVD, chronic lower respiratory tract diseases, Alzheimer’s disease, and influenza and pneumonia, whereas heavy or binge drinking were associated with a significantly higher risk of mortality from all causes, cancer, and accidents (unintentional injuries). Light or moderate alcohol consumption was also inversely associated with mortality from diabetes mellitus and nephritis, nephrotic syndrome, or nephrosis. The protective effect of light to moderate alcohol consumption was more pronounced in women, older populations, non-Hispanic white subjects, and never smokers, whereas the adverse effect of heavy drinking on mortality from all causes, cancer, and accidents (unintentional injuries) was obvious in younger age groups and those who ever smoked.

Alcohol use has a complex association with health. Our study suggested that heavy alcohol consumption was associated with a significantly higher risk of mortality from all causes, cancer, and accidents (unintentional injuries). The results are consistent with a recent study showed that there were the strong association of alcohol use with the risk of cancer and injuries [1]. These indicate that mortality from cancer and accidents (unintentional injuries) may be the main causes of death caused by alcohol consumption.

Our findings of the associations between alcohol consumption and all-cause, CVD, and cancer mortality were consistent with some [3, 4, 12, 13, 46, 47], but not all [6, 7, 27, 48] previous studies. Analyses of higher-quality studies free from abstainer biases found no evidence of reduced risk of mortality at low levels of alcohol consumption [6]. Some studies on alcohol and health may misclassify former and occasional drinkers as abstainers and place them in the reference group, which may underlie positive health outcomes observed in people with low alcohol consumption. Considering abstainer biases, former or current infrequent drinkers were not included in the “abstainer” reference group, which may provide more accurate information on the drinker category in the current study. Compared with the two previous studies on the NHIS [12, 13], this study also included more recent data and three additional category groups: “former infrequent drinker,” “former regular drinker,” and “current infrequent drinker.” This study found beneficial effects of current infrequent alcohol consumption on all-cause and CVD mortality, which were not found in the two previous studies [12, 13]. The current study has incorporated updated data from NHIS spanning from 1997 to 2014, along with mortality outcomes that were tracked until the end of 2019. In addition, this study examined the association between alcohol consumption and cause-specific mortality, such as mortality from chronic lower respiratory tract diseases, accidents (unintentional injuries), Alzheimer’s disease, diabetes mellitus, influenza and pneumonia, and nephritis, nephrotic syndrome, or nephrosis.

Stratified analyses by sex suggested that a protective effect of light or moderate drinking on mortality was lower in men than in women and current heavy drinkers had a higher risk of all-cause and cancer mortality in men but not in women in this study. One study in China found that alcohol consumption was associated with a lower risk of COPD mortality in men than in women [49]. Data from the nationwide China Kadoorie Biobank prospective study showed that men drink more than 20 times as much as women [50]. Genetic evidence shows that the apparently protective effects of moderate alcohol intake against stroke are not mainly caused by alcohol itself and are largely artifacts of reverse causation and confounding [50]. Alcohol drinking remains predominately a male phenomenon in China, and distilled spirits are the main type of alcoholic beverage, which is very different from that in most Western populations. The current study of the US population observed a protective effect of light drinking on mortality from chronic lower respiratory tract diseases. Previous studies also found an association between moderate drinking and a lower respiratory disease mortality rate [51, 52]. A cohort study of participants aged 65 years or older found that occasional or moderate drinking was associated with a lower risk of death from all respiratory disease and COPD [53]. A cross-sectional study of men from Finland, Italy, and the Netherlands showed that moderate drinkers had lower COPD mortality relative to nondrinkers and heavy drinkers [54]. It is possible that mild concentrations of alcohol increase mucociliary clearance and bronchodilation and reduce the airway inflammation and injury found in asthma and COPD [55]. On the other hand, prolonged and heavy drinking may impair mucociliary clearance, worsen asthma, and most likely lead to lung-function decline and mortality in patients with COPD [55].

Most studies that estimate the risk of alcohol-related injuries primarily focus on data from hospital emergency departments [56], so the risk of alcohol-related injuries may be underestimated. In one study, the estimates of emergency department injury ranged from 5 to 40% by using emergency department data from 27 countries [57]. This prospective population-based study found that heavy drinkers had a 71% higher risk of mortality accidents (unintentional injuries) after excluding those with chronic diseases. A study indicated strong dose–response associations of amount of alcohol consumption in the past 3 h with odds of vehicle injury [58]. A meta-analysis showed that even moderate alcohol consumption roughly doubled the odds of injury and that the risks increased sharply at higher levels of alcohol intake [56]. Overall, this study concludes that actions to reduce the risk of death from accidents (unintentional injuries) associated with alcohol consumption, especially heavy drinking patterns, should be urgently strengthened.

This study found a favorable effect of current infrequent, light, or moderate drinking on mortality from Alzheimer’s disease in people older than 60 but not in those younger than 60. One study concluded that the risk of dementia-related death was significantly higher among elderly abstainers compared to individuals that drank alcohol [29], which was consistent with our findings. A Mendelian randomization study found a causal association between alcohol consumption and an earlier Alzheimer’s disease age of onset survival, but not between alcohol consumption and late-onset Alzheimer’s disease risk [59]. Two meta-analyses found that light to moderate alcohol consumption was associated with a 25–38% reduction in the risk of Alzheimer’s disease, vascular dementia, and all-cause dementia compared with abstainers [30, 31]. Our findings indicated that current infrequent, light, or moderate drinkers had a 17–32% lower risk of mortality due to Alzheimer’s disease compared to lifetime abstainers. However, this study cannot determine the causal relationship between alcohol consumption and Alzheimer’s disease mortality. Light or moderate alcohol use is probably not healthy by itself, but it is a marker of other healthy habits. It is possible that light or moderate alcohol use is just something that healthy people do. The molecular mechanisms of Alzheimer’s disease are still not entirely clear.

The current study using the NHIS data strictly defined a non-drinking category, in which former drinkers were not included in the reference group. Our results suggested that light or moderate drinkers had a lower risk of diabetes mortality. One meta-analysis showed no reduction in the risk of type 2 diabetes at any level of alcohol consumption among men and reductions in risk among moderate alcohol drinkers being specific to women [60]. However, Joosten et al. studied 35,625 adults aged 20–70 years and found that moderate alcohol consumption was associated with an approximately 40% lower risk of type 2 diabetes compared with abstention, and they did not find differences between the two sexes [61]. Given that no clear conclusion has been reached, recommendations must be made cautiously, and the association between drinking alcohol and diabetes mortality is still a research topic.

Our study found that current infrequent, light, or moderate drinking were associated with a lower risk of mortality from influenza and pneumonia. A prospective Chinese elderly cohort study also found a lower risk of pneumonia mortality in occasional drinkers [53]. Clinical studies [62, 63] have observed a beneficial effect of alcohol level on pneumonia rate in patients with traumatic brain injury, although the exact mechanism of the effect is unknown. However, one meta-analysis suggested an 83% increased risk of community-acquired pneumonia among adults who consumed some or high amounts of alcohol compared to those who consumed no or low amounts [64]. Thus, further research is needed to confirm the association between alcohol use and the risk of mortality from influenza and pneumonia.

This large cohort study found that light or moderate drinkers were associated with a lower risk of mortality from nephritis, nephrotic syndrome, or nephrosis. One study of an elderly Italian population suggested that moderate quantities of alcohol were not injurious to renal function [65]. Nevertheless, another study of rat models of acute and chronic progressive anti-thy1 glomerulonephritis suggested that moderate alcohol consumption might not bring specific protection in renal fibrotic disease [66]. Alcohol consumption has been related to the development or progression of chronic kidney disease [67]. A prospective population-based study found that moderate-heavy drinking was associated with elevated risk of albuminuria compared to light drinking [68]. It is likely that the effect of alcohol consumption on the risk of mortality from kidney diseases depends on the level of alcohol consumption. Nonetheless, due to the complexity of the pathogenesis of nephritis, nephrotic syndrome, or nephrosis, its causes and mechanism is still debatable. Therefore, more research is necessary to gain further knowledge on this topic.

We acknowledge that there are several limitations of the current study. First, as in other observational studies, our findings might be confounded by unidentified confounders that were not fully adjusted for, although there is relatively wide range of covariates available in NHIS. Second, the assessment of self-reported alcohol consumption in the NHIS was conducted at a single point in time, and it is possible that the research participants modified their consumption behavior during follow-up. Third, information on specific types of alcoholic beverages consumed was not collected uniformly. The effects of other components of each type of alcoholic drink besides ethanol on mortality risk cannot be fully ruled out. Fourth, the estimates may have been influenced by selection and misclassification biases of different participants; thus, we conducted sensitivity analyses by excluding participants who died in the first 2 years and those with physician-diagnosed diseases. Although the results of this study were basically consistent, these methods may not be sufficient to solve this problem. Fifth, due to stratifying participants by several obvious confounding factors and drinking habits simultaneously, the power may be insufficient to accurately estimate the risk of drinking, although the overall sample size is relatively large in the current study. Sixth, this study cannot distinguish mortality from specific cancer types. The findings of cancer mortality may not align with cause-specific cancer outcomes, such as liver cancer, oral cancer, and esophageal cancer. Also, the results related to cancer mortality may differ from the findings related to cancer incidence. Seventh, light or moderate drinking may provide some benefits in preventing deaths due to CVD, chronic lower respiratory tract diseases, Alzheimer’s disease, diabetes mellitus, influenza and pneumonia, and nephritis, nephrotic syndrome, or nephrosis, but this study cannot determine the causal relationship between alcohol consumption and those causes of death. Finally, the NHIS Linked Mortality File identified causes of death information by linkage to the NDI, which is derived from death certificates. Although this methodology has been previously validated by many published reports, the possibility of cause-of-death misclassification cannot be ruled out.

## Conclusions

This large prospective study of US adults indicated that infrequent, light, or moderate drinking were associated with a lower risk of mortality from all causes, CVD, chronic lower respiratory tract diseases, Alzheimer’s disease, and influenza and pneumonia. Light or moderate drinking might have a protective effect on mortality from diabetes mellitus and nephritis, nephrotic syndrome, or nephrosis. However, heavy or binge drinking were associated with a significantly higher risk of all-cause, cancer, and accident (unintentional injuries) mortality. The deleterious effect of heavy alcohol consumption was apparent, although the beneficial effects of lower consumption were still observed. Therefore, recommending drinking must be done with caution.

## Supplementary Information

**Additional file 1: **Supplementary Materials. ICD-10 codes for causesof death used in this study. Flow chart of the selection of study participants. The distribution of alcohol consumption accordingto NHIS year among NHIS participants in 1997 to 2014.Dose-response relationship between alcohol consumption and risk ofmortality from CVD, chronic lower respiratory tract diseases, accidents, Alzheimer’s disease, diabetes mellitus, influenzaand pneumonia, and nephritis, nephrotic syndrome, or nephrosis mortality. All-cause and cause-specific mortality according to alcoholconsumption status after multiple imputations for variables with missingvalues among NHIS participants in 1997to 2014. All-cause andcause-specific mortalityaccording to alcohol consumption status among NHIS participants in 1997to 2014. All-cause mortality according to alcohol consumptionstatus and NHIS year among NHIS participants in 1997 to2014.Hazards ratios for all-cause and cause-specific mortality according to alcoholconsumption status among NHIS participants in 1997 to 2014. Hazards ratios forall-cause and cause-specific mortality according to alcohol consumptionstatus stratified for sex, age, race/ethnicity and smoking status amongNHIS participants in 1997 to 2014.

## Data Availability

The NHIS data (www.cdc.gov/nchs/nhis/index.htm) are available to researchers upon application.

## Abbreviations

CI: Confidence interval
COPD: Obstructive pulmonary disease
CVD: Cardiovascular disease
HR: Hazard ratio
ICD-10: International Statistical Classification of Diseases, Injuries, and Causes of Death
NCHS: National Center for Health Statistics
NDI: National Death Index
NHIS: National Health Interview Survey
