# Supplementary material for: Alcohol consumption and all-cause and cause-specific mortality among US adults: prospective cohort study
Source: BMC Med. 2023 Jun 7;21:208. doi: 10.1186/s12916-023-02907-6 (PMC10249162; doi:10.1186/s12916-023-02907-6)
Supplement: Supplementary file 1 — Additional file 1: Supplementary Materials. ICD-10 codes for causesof death used in this study. Flow chart of the selection of study participants. The distribution of alcohol consumption accordingto NHIS year among NHIS participants in 1997 to 2014.Dose-response relationship between alcohol consumption and risk ofmortality from CVD, chronic lower respiratory tract diseases, accidents, Alzheimer’s disease, diabetes mellitus, influenzaand pneumonia, and nephritis, nephrotic syndrome, or nephrosis mortality. All-cause and cause-specific mortality according to alcoholconsumption status after multiple imputations for variables with missingvalues among NHIS participants in 1997to 2014. All-cause andcause-specific mortalityaccording to alcohol consumption status among NHIS participants in 1997to 2014. All-cause mortality according to alcohol consumptionstatus and NHIS year among NHIS participants in 1997 to2014.Hazards ratios for all-cause and cause-specific mortality according to alcoholconsumption status among NHIS participants in 1997 to 2014. Hazards ratios forall-cause and cause-specific mortality according to alcohol consumptionstatus stratified for sex, age, race/ethnicity and smoking status amongNHIS participants in 1997 to 2014. [file 12916_2023_2907_MOESM1_ESM.doc]

**SUPPLEMENTAL MATERIAL**

**Table S1.** ICD-10 codes for causes of death used in this study

**Figure S1.** Flow chart of the selection of study participants

**Table S2.** The distribution of alcohol consumption according to NHIS year among NHIS participants in 1997 to 2014

**Figure S2.** Dose-response relationship between alcohol consumption and risk of mortality from CVD

**Figure S3.** Dose-response relationship between alcohol consumption and risk of mortality from cancer

**Figure S4.** Dose-response relationship between alcohol consumption and risk of mortality from chronic lower respiratory tract diseases

**Figure S5.** Dose-response relationship between alcohol consumption and risk of mortality from accidents (unintentional injuries)

**Figure S6.** Dose-response relationship between alcohol consumption and risk of mortality from Alzheimer’s disease

**Figure S7.** Dose-response relationship between alcohol consumption and risk of mortality from diabetes mellitus

**Figure S8.** Dose-response relationship between alcohol consumption and risk of mortality from influenza and pneumonia

**Figure S9.** Dose-response relationship between alcohol consumption and risk of mortality from nephritis, nephrotic syndrome, or nephrosis

**Table S3.** All-cause and cause-specific mortality according to alcohol consumption status after multiple imputations for variables with missing values among NHIS participants in 1997 to 2014

**Table S4.** All-cause and cause-specific mortality (current infrequent drinkers as reference group) according to alcohol consumption status among NHIS participants in 1997 to 2014

**Table S5.** All-cause mortality according to alcohol consumption status and NHIS year among NHIS participants in 1997 to 2014

**Table S6.** Hazards ratios for all-cause and cause-specific mortality according to alcohol consumption status among NHIS participants in 1997 to 2014 (excluding participants with heart disease, stroke, cancer, diabetes, hypertension, asthma, emphysema, and chronic bronchitis)

**Table S7.** Hazards ratios for all-cause and cause-specific mortality according to alcohol consumption status stratified for sex, age, race/ethnicity and smoking status among NHIS participants in 1997 to 2014

| **Table S1.** ICD-10 codes for causes of death used in this study |
| --- |
| Cardiovascular disease—I00-I09, I11, I13, I20-I51, I60-I69 |
| Cancer—C00-C97 |
| Chronic lower respiratory tract diseases—J40-J47 |
| Accidents and injuries—V01-X59, Y85-Y86 |
| Alzheimer’s disease—G30 |
| Diabetes—E10-E14 |
| Influenza and pneumonia—J09-18 |
| Nephritis, nephrotic syndrome, or nephrosis—N00-N07, N17-N19, N25-N27 |
| ICD-10: international classification of diseases, 10th revision |

**Figure S1.** Flow chart of the selection of study participants

| **Table S2.** The distribution of alcohol consumption according to NHIS survey year among NHIS participants in 1997 to 2014 | | | | | | | | |
| --- | --- | --- | --- | --- | --- | --- | --- | --- |
| **Survey Year** | **Lifetime abstainer** | **Former infrequent drinker** | **Former regular drinker** | **Current infrequent drinker** | **Current light drinker** | **Current moderate drinker** | **Current heavy drinker** | **Overall** |
| 1997 | 13910 (23.14) | - | 9563 (15.91) | 8449 (14.05) | 17425 (28.98) | 7372 (12.26) | 3404 (5.66) | 60123 (6.55) |
| 1998 | 12381 (23.38) | - | 8776 (16.57) | 7267 (13.72) | 15135 (28.58) | 6567 (12.40) | 2832 (5.35) | 52958 (5.77) |
| 1999 | 12436 (24.79) | - | 7785 (15.52) | 6683 (13.32) | 14349 (28.61) | 6184 (12.33) | 2724 (5.43) | 50161 (5.46) |
| 2000 | 13852 (26.10) | - | 7879 (14.84) | 7244 (13.65) | 15263 (28.75) | 6113 (11.52) | 2729 (5.14) | 53080 (5.78) |
| 2001 | 13285 (24.60) | 4729 (8.76) | 3468 (6.42) | 7131 (13.21) | 15610 (28.91) | 7264 (13.45) | 2510 (4.65) | 53997 (5.88) |
| 2002 | 12126 (24.23) | 4341 (8.68) | 3460 (6.91) | 6766 (13.53) | 14261 (28.50) | 6707 (13.40) | 2377 (4.75) | 50038 (5.45) |
| 2003 | 12866 (26.44) | 3951 (8.12) | 3170 (6.51) | 6473 (13.30) | 13496 (27.73) | 6501 (13.36) | 2210 (4.54) | 48667 (5.30) |
| 2004 | 13282 (26.48) | 4222 (8.42) | 3364 (6.71) | 6694 (13.35) | 13896 (27.71) | 6573 (13.11) | 2124 (4.23) | 50155 (5.46) |
| 2005 | 12786 (25.58) | 4283 (8.57) | 3259 (6.52) | 6226 (12.46) | 14251 (28.51) | 6874 (13.75) | 2305 (4.61) | 49984 (5.44) |
| 2006 | 11213 (27.73) | 3391 (8.39) | 2560 (6.33) | 5062 (12.52) | 11024 (27.26) | 5390 (13.33) | 1797 (4.44) | 40437 (4.40) |
| 2007 | 10226 (26.52) | 3389 (8.79) | 2611 (6.77) | 4942 (12.82) | 10700 (27.75) | 4956 (12.85) | 1731 (4.49) | 38555 (4.20) |
| 2008 | 8685 (23.63) | 3342 (9.09) | 2200 (5.99) | 5071 (13.80) | 10517 (28.62) | 5049 (13.74) | 1884 (5.13) | 36748 (4.00) |
| 2009 | 11024 (22.88) | 4579 (9.50) | 3003 (6.23) | 6360 (13.20) | 14163 (29.40) | 6796 (14.11) | 2254 (4.68) | 48179 (5.25) |
| 2010 | 11347 (23.94) | 4325 (9.12) | 2825 (5.96) | 6511 (13.74) | 13785 (29.08) | 6418 (13.54) | 2192 (4.62) | 47403 (5.16) |
| 2011 | 12835 (22.44) | 5406 (9.46) | 3493 (6.11) | 7849 (13.73) | 16978 (29.70) | 8408 (14.08) | 2561 (4.48) | 57160 (6.22) |
| 2012 | 13787 (23.30) | 5347 (9.04) | 3850 (6.51) | 7686 (12.99) | 17368 (29.36) | 8335 (14.09) | 2791 (4.72) | 59164 (6.44) |
| 2013 | 13557 (23.03) | 5398 (9.17) | 3570 (6.06) | 7756 (13.17) | 17205 (29/22) | 8444 (14.34) | 2941 (5.00) | 58871 (6.41) |
| 2014 | 14169 (22.54) | 5482 (8.72) | 3933 (6.26) | 8271 (13.16) | 18641 (29.66) | 9234 (14.69) | 3119 (4.96) | 62849 (6.84) |
| 1997-2014 | 223757(24.36) | 62185 (6.77) | 78769 (8.58) | 122441 (13.33) | 264067 (28.75) | 122825 (13.37) | 44485 (4.84) | 918529 (100.00) |
| Values are numbers (percentages) unless stated otherwise | | | | | | | | |

**Figure S2.** Dose-response relationship between alcohol consumption and risk of mortality from CVD. A nonlinear relationship of current alcohol consumption (as a continuous variable) with CVD mortality (p < 0.05 for the nonlinear test), using maximally adjusted estimates (adjusted for sex, age, race/ethnicity, education, marital status, body mass index, physical activity, smoking, and physician-diagnosed diseases (heart disease, stroke, cancer, diabetes, hypertension, asthma, emphysema, and chronic bronchitis). CVD = cardiovascular disease, HR = hazard ratio.


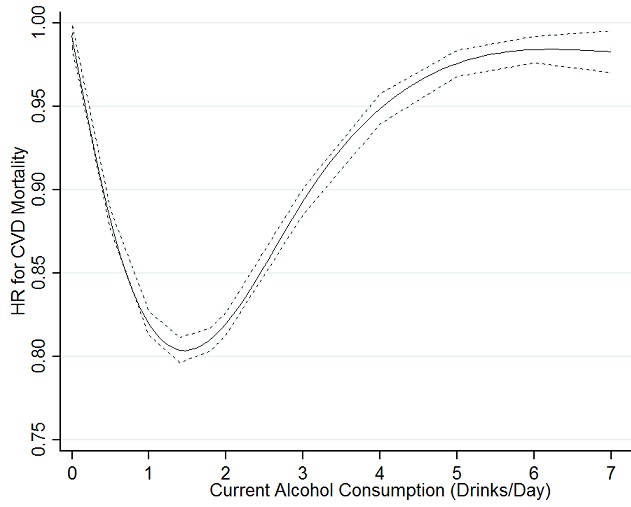


**Figure S3.** Dose-response relationship between alcohol consumption and risk of mortality from cancer. A nonlinear relationship of current alcohol consumption (as a continuous variable) with cancer mortality (p < 0.05 for the nonlinear test), using maximally adjusted estimates (adjusted for sex, age, race/ethnicity, education, marital status, body mass index, physical activity, smoking, and physician-diagnosed diseases (heart disease, stroke, cancer, diabetes, hypertension, asthma, emphysema, and chronic bronchitis). HR = hazard ratio.


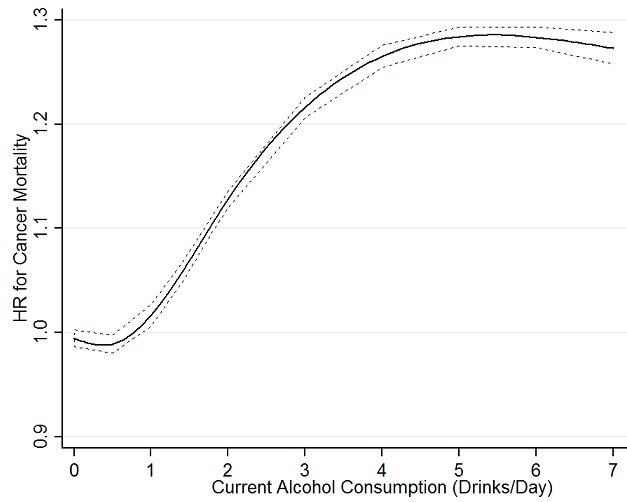


**Figure S4.** Dose-response relationship between alcohol consumption and risk of mortality from chronic lower respiratory tract diseases. A nonlinear relationship of current alcohol consumption (as a continuous variable) with chronic lower respiratory tract diseases mortality (p < 0.05 for the nonlinear test), using maximally adjusted estimates (adjusted for sex, age, race/ethnicity, education, marital status, body mass index, physical activity, smoking, and physician-diagnosed diseases (heart disease, stroke, cancer, diabetes, hypertension, asthma, emphysema, and chronic bronchitis). HR = hazard ratio.


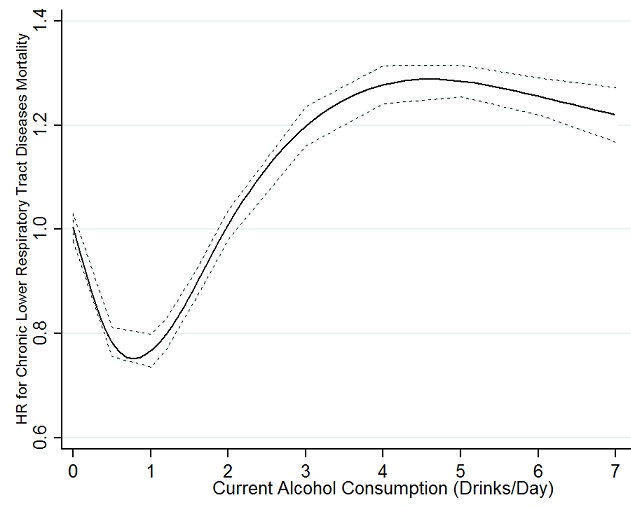


**Figure S5.** Dose-response relationship between alcohol consumption and risk of mortality from accidents (unintentional injuries). A nonlinear relationship of current alcohol consumption (as a continuous variable) with accidents (unintentional injuries) mortality (p < 0.05 for the nonlinear test), using maximally adjusted estimates (adjusted for sex, age, race/ethnicity, education, marital status, body mass index, physical activity, smoking, and physician-diagnosed diseases (heart disease, stroke, cancer, diabetes, hypertension, asthma, emphysema, and chronic bronchitis). HR = hazard ratio.


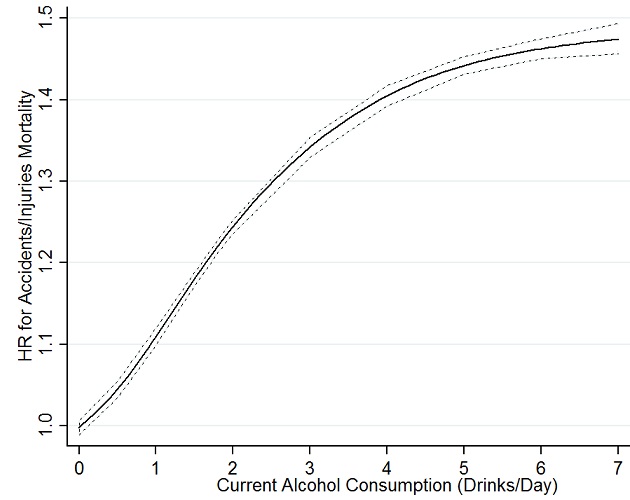


**Figure S6.** Dose-response relationship between alcohol consumption and risk of mortality from Alzheimer’s disease. A nonlinear relationship of current alcohol consumption (as a continuous variable) with Alzheimer’s disease mortality (p < 0.05 for the nonlinear test), using maximally adjusted estimates (adjusted for sex, age, race/ethnicity, education, marital status, body mass index, physical activity, smoking, and physician-diagnosed diseases (heart disease, stroke, cancer, diabetes, hypertension, asthma, emphysema, and chronic bronchitis). HR = hazard ratio.


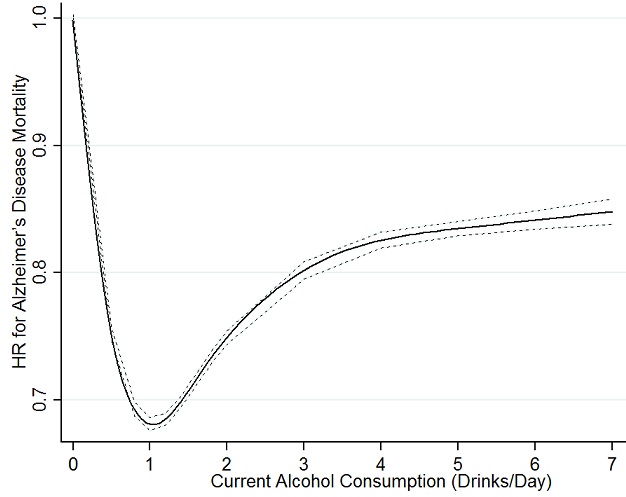


**Figure S7.** Dose-response relationship between alcohol consumption and risk of mortality from diabetes mellitus. A nonlinear relationship of current alcohol consumption (as a continuous variable) with diabetes mortality (p < 0.05 for the nonlinear test), using maximally adjusted estimates (adjusted for sex, age, race/ethnicity, education, marital status, body mass index, physical activity, smoking, and physician-diagnosed diseases (heart disease, stroke, cancer, diabetes, hypertension, asthma, emphysema, and chronic bronchitis). HR = hazard ratio.


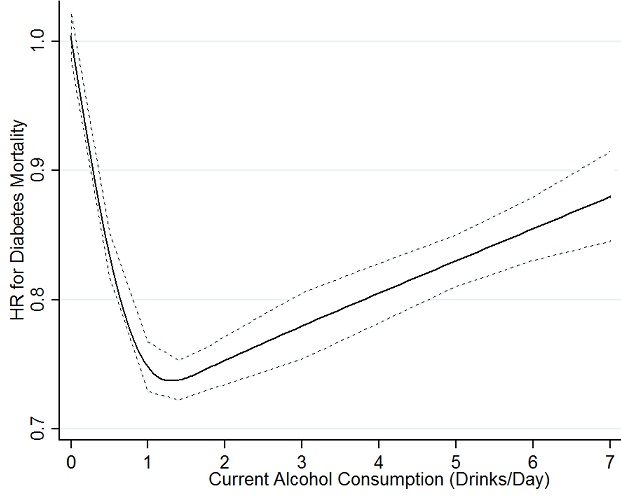


**Figure S8.** Dose-response relationship between alcohol consumption and risk of mortality from influenza and pneumonia. A nonlinear relationship of current alcohol consumption (as a continuous variable) with influenza and pneumonia mortality (p < 0.05 for the nonlinear test), using maximally adjusted estimates (adjusted for sex, age, race/ethnicity, education, marital status, body mass index, physical activity, smoking, and physician-diagnosed diseases (heart disease, stroke, cancer, diabetes, hypertension, asthma, emphysema, and chronic bronchitis). HR = hazard ratio.


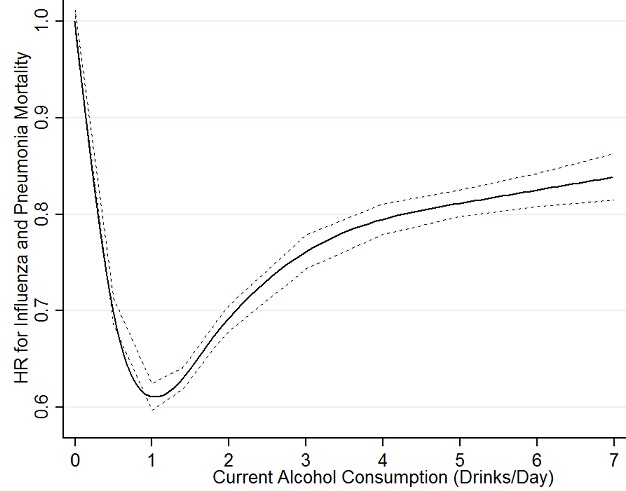


**Figure S9.** Dose-response relationship between alcohol consumption and risk of mortality from nephritis, nephrotic syndrome, or nephrosis. A nonlinear relationship of current alcohol consumption (as a continuous variable) with nephritis, nephrotic syndrome, or nephrosis mortality (p < 0.05 for the nonlinear test), using maximally adjusted estimates (adjusted for sex, age, race/ethnicity, education, marital status, body mass index, physical activity, smoking, and physician-diagnosed diseases (heart disease, stroke, cancer, diabetes, hypertension, asthma, emphysema, and chronic bronchitis). HR = hazard ratio.


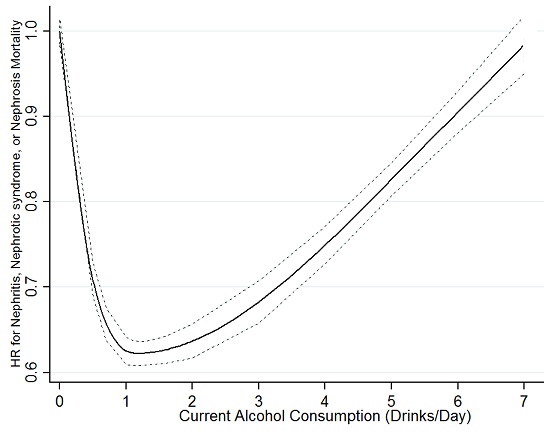


| **Table S3.** All-cause and cause-specific mortality according to alcohol consumption status after multiple imputations for variables with missing values among NHIS participants in 1997 to 2014 | | | | | | | | | | | | | | |  |
| --- | --- | --- | --- | --- | --- | --- | --- | --- | --- | --- | --- | --- | --- | --- | --- |
|  | | **Lifetime abstainer** | **Former infrequent drinker** | | **Former regular drinker** | | **Current infrequent drinker** | | **Current light drinker** | | **Current moderate drinker** | | **Current heavy drinker** | |  |
| All-cause | |  |  | |  | |  | |  | |  | |  | |  |
| Model 1 | | 1 (ref) | 1.02 (0.99–1.04) | | 1.06 (1.04–1.09) | | 0.91 (0.89–0.93) | | 0.81 (0.79–0.83) | | 0.84 (0.82–0.86) | | 1.03 (1.00–1.06) | |  |
| CVD | |  |  | |  | |  | |  | |  | |  | |  |
| Model 1 | | 1 (ref) | 0.99 (0.95–1.03) | | 1.05 (1.02–1.09) | | 0.91 (0.88–0.94) | | 0.81 (0.78–0.84) | | 0.80 (0.77–0.84) | | 0.93 (0.88–1.00) | |  |
| Cancer | |  |  | |  | |  | |  | |  | |  | |  |
| Model 1 | | 1 (ref) | 1.04 (0.99–1.09) | | 1.10 (1.05–1.16) | | 0.95 (0.91–1.00) | | 0.86 (0.83–0.90) | | 0.91 (0.87–0.96) | | 1.15 (1.08–1.22) | |  |
| Chronic lower respiratory tract diseases | |  |  | |  | |  | |  | |  | |  | |  |
| Model 1 | | 1 (ref) | 1.01 (0.93–1.11) | | 1.07 (0.99–1.16) | | 0.91 (0.83–0.99) | | 0.69 (0.63–0.76) | | 0.76 (0.69–0.84) | | 0.92 (0.82–1.03) | |  |
| Accidents and injuries | |  |  | |  | |  | |  | |  | |  | |  |
| Model 1 | | 1 (ref) | 1.10 (0.96–1.25) | | 1.03 (0.92–1.15) | | 0.93 (0.83–1.04) | | 0.90 (0.82–1.00) | | 0.87 (0.78–0.99) | | 1.27 (1.09–1.47) | |  |
| Alzheimer’s disease | |  |  | |  | |  | |  | |  | |  | |  |
| Model 1 | | 1 (ref) | 1.14 (1.00–1.29) | | 0.94 (0.84–1.05) | | 0.86 (0.76–0.96) | | 0.80 (0.72–0.89) | | 0.85 (0.74–0.96) | | 0.86 (0.70–1.05) | |  |
| Diabetes mellitus | |  |  | |  | |  | |  | |  | |  | |  |
| Model 1 | | 1 (ref) | 0.97 (0.87–1.10) | | 1.12 (1.01–1.24) | | 0.93 (0.83–1.05) | | 0.78 (0.69–0.88) | | 0.74 (0.64–0.85) | | 0.87 (0.70–1.08) | |  |
| Influenza and pneumonia | |  |  | |  | |  | |  | |  | |  | |  |
| Model 1 | | 1 (ref) | 0.94 (0.80–1.09) | | 1.06 (0.93–1.21) | | 0.83 (0.72–0.96) | | 0.76 (0.66–0.87) | | 0.68 (0.57–0.81) | | 0.98 (0.77–1.24) | |  |
| Nephritis, nephrotic syndrome, or nephrosis | |  |  | |  | |  | |  | |  | |  | |  |
| Model 1 | | 1 (ref) | 1.00 (0.86–1.16) | | 0.97 (0.85–1.11) | | 0.92 (0.80–1.07) | | 0.76 (0.65–0.88) | | 0.66 (0.55–0.80) | | 0.99 (0.76–1.27) | |  |
| Model 1: Adjusted for sex, age, race/ethnicity, education, marital status, body mass index, smoking status, physical activity, and chronic conditions (heart disease, stroke, cancer, diabetes, hypertension, asthma, emphysema, and chronic bronchitis) | | | | | | | | | | | | | | |  |
| **Table S4.** All-cause and cause-specific mortality (current infrequent drinkers as reference group) according to alcohol consumption status among NHIS participants in 1997 to 2014 | | | | | | | | | | | | | | | |
|  | **Lifetime abstainer** | | | **Former infrequent drinker** | | **Former regular drinker** | | **Current infrequent drinker** | | **Current light drinker** | | **Current moderate drinker** | | **Current heavy drinker** | |
| All-cause |  | | |  | |  | |  | |  | |  | |  | |
| Model 1 | 1.15 (1.12–1.18) | | | 1.15 (1.11–1.19) | | 1.24 (1.21–1.28) | | 1 (ref) | | 0.89 (0.86–0.91) | | 0.95 (0.91–0.98) | | 1.23 (1.18–1.29) | |
| CVD |  | | |  | |  | |  | |  | |  | |  | |
| Model 1 | 1.16 (1.10–1.22) | | | 1.13 (1.05–1.21) | | 1.25 (1.18–1.32) | | 1 (ref) | | 0.88 (0.83–0.93) | | 0.90 (0.85–0.96) | | 1.08 (0.99–1.18) | |
| Cancer |  | | |  | |  | |  | |  | |  | |  | |
| Model 1 | 1.05 (0.99–1.12) | | | 1.10 (1.03–1.19) | | 1.20 (1.13–1.28) | | 1 (ref) | | 0.90 (0.85–0.96) | | 0.97 (0.91–1.04) | | 1.30 (1.20–1.41) | |
| Chronic lower respiratory tract diseases |  | | |  | |  | |  | |  | |  | |  | |
| Model 1 | 1.14 (1.01–1.29) | | | 1.13 (0.98–1.31) | | 1.24 (1.09–1.41) | | 1 (ref) | | 0.77 (0.68–0.88) | | 0.89 (0.77–1.04) | | 1.08 (0.93–1.26) | |
| Accidents and injuries |  | | |  | |  | |  | |  | |  | |  | |
| Model 1 | 1.18 (1.01–1.39) | | | 1.27 (1.03–1.58) | | 1.23 (1.02–1.47) | | 1 (ref) | | 1.13 (0.96–1.33) | | 1.06 (0.89–1.28) | | 1.75 (1.41–2.19) | |
| Alzheimer’s disease |  | | |  | |  | |  | |  | |  | |  | |
| Model 1 | 1.32 (1.13–1.54) | | | 1.39 (1.15–1.68) | | 1.25 (1.06–1.48) | | 1 (ref) | | 0.89 (0.75–1.07) | | 1.09 (0.90–1.32) | | 0.86 (0.61–1.22) | |
| Diabetes mellitus |  | | |  | |  | |  | |  | |  | |  | |
| Model 1 | 1.09 (0.93–1.27) | | | 1.00 (0.83–1.19) | | 1.27 (1.08–1.48) | | 1 (ref) | | 0.78 (0.65–0.93) | | 0.79 (0.63–0.98) | | 1.01 (0.72–1.40) | |
| Influenza and pneumonia |  | | |  | |  | |  | |  | |  | |  | |
| Model 1 | 1.46 (1.20–1.76) | | | 1.07 (0.83–1.38) | | 1.42 (1.14–1.77) | | 1 (ref) | | 0.91 (0.73–1.14) | | 0.84 (0.65–1.10) | | 1.17 (0.82–1.66) | |
| Nephritis, nephrotic syndrome, or nephrosis |  | | |  | |  | |  | |  | |  | |  | |
| Model 1 | 0.99 (0.81–1.20) | | | 0.96 (0.76–1.22) | | 0.93 (0.75–1.16) | | 1 (ref) | | 0.65 (0.52–0.82) | | 0.61 (0.46–0.79) | | 0.97 (0.69–1.36) | |
| Model 1: Adjusted for sex, age, race/ethnicity, education, marital status, body mass index, smoking status, physical activity, and chronic conditions (heart disease, stroke, cancer, diabetes, hypertension, asthma, emphysema, and chronic bronchitis) | | | | | | | | | | | | | | | |

| **Table S5.** All-cause mortality according to alcohol consumption status and NHIS year among NHIS participants in 1997 to 2014 | | | | | | | |
| --- | --- | --- | --- | --- | --- | --- | --- |
| **Survey Year** | **Lifetime abstainer** | **Former infrequent drinker** | **Former regular drinker** | **Current infrequent drinker** | **Current light drinker** | **Current moderate drinker** | **Current heavy drinker** |
| 1997 | 1 (ref) | - | 1.09 (1.01–1.17) | 0.82 (0.75–0.89) | 0.80 (0.74–0.86) | 0.84 (0.78–0.91) | 1.21 (1.08–1.36) |
| 1998 | 1 (ref) | - | 1.05 (0.97–1.14) | 0.84 (0.77–0.92) | 0.85 (0.78–0.93) | 0.88 (0.79–0.97) | 1.19 (1.04–1.36) |
| 1999 | 1 (ref) | - | 1.05 (0.98–1.13) | 0.84 (0.76–0.93) | 0.77 (0.70–0.84) | 0.85 (0.77–0.94) | 1.05 (0.92–1.21) |
| 2000 | 1 (ref) | - | 1.07 (0.99–1.16) | 0.95 (0.87–1.05) | 0.84 (0.76–0.92) | 0.88 (0.79–0.98) | 1.09 (0.94–1.26) |
| 2001 | 1 (ref) | 1.02 (0.92–1.12) | 1.10 (0.99–1.23) | 0.88 (0.80–0.96) | 0.80 (0.73–0.88) | 0.89 (0.80–0.99) | 1.04 (0.90–1.21) |
| 2002 | 1 (ref) | 0.95 (0.87–1.05) | 1.02 (0.91–1.15) | 0.85 (0.77–0.95) | 0.77 (0.70–0.84) | 0.79 (0.70–0.88) | 1.18 (1.02–1.36) |
| 2003 | 1 (ref) | 0.97 (0.86–1.08) | 1.06 (0.93–1.19) | 0.88 (0.79–0.98) | 0.76 (0.69–0.85) | 0.81 (0.72–0.92) | 1.00 (0.85–1.17) |
| 2004 | 1 (ref) | 0.99 (0.89–1.09) | 1.08 (0.98–1.21) | 0.89 (0.80–0.99) | 0.80 (0.72–0.88) | 0.84 (0.75–0.95) | 1.26 (1.06–1.49) |
| 2005 | 1 (ref) | 0.92 (0.83–1.03) | 0.98 (0.87–1.11) | 0.81 (0.72–0.91) | 0.72 (0.65–0.80) | 0.83 (0.74–0.93) | 1.00 (0.85–1.17) |
| 2006 | 1 (ref) | 1.02 (0.90–1.16) | 0.99 (0.86–1.14) | 0.85 (0.74–0.98) | 0.75 (0.66–0.85) | 0.83 (0.71–0.97) | 1.17 (0.95–1.42) |
| 2007 | 1 (ref) | 1.17 (1.01–1.35) | 1.19 (1.01–1.40) | 0.97 (0.84–1.13) | 0.78 (0.67–0.90) | 0.77 (0.66–0.91) | 1.19 (0.91–1.54) |
| 2008 | 1 (ref) | 1.03 (0.88–1.20) | 1.00 (0.86–1.18) | 0.83 (0.70–0.98) | 0.73 (0.62–0.86) | 0.85 (0.71–1.02) | 1.10 (0.86–1.40) |
| 2009 | 1 (ref) | 1.07 (0.94–1.23) | 1.12 (0.96–1.30) | 0.86 (0.74–0.99) | 0.70 (0.61–0.81) | 0.81 (0.70–0.96) | 1.02 (0.82–1.25) |
| 2010 | 1 (ref) | 1.12 (0.98–1.27) | 1.21 (1.04–1.41) | 0.90 (0.76–1.06) | 0.75 (0.64–0.86) | 0.83 (0.70–0.98) | 1.03 (0.82–1.29) |
| 2011 | 1 (ref) | 0.96 (0.83–1.11) | 1.08 (0.93–1.26) | 0.91 (0.79–1.06) | 0.75 (0.66–0.86) | 0.76 (0.65–0.89) | 0.75 (0.59–0.95) |
| 2012 | 1 (ref) | 0.93 (0.80–1.08) | 1.06 (0.91–1.24) | 0.98 (0.83–1.15) | 0.63 (0.54–0.73) | 0.67 (0.55–0.82) | 0.93 (0.75–1.16) |
| 2013 | 1 (ref) | 1.03 (0.86–1.22) | 1.06 (0.89–1.28) | 0.87 (0.72–1.05) | 0.79 (0.67–0.95) | 0.77 (0.63–0.94) | 0.83 (0.61–1.11) |
| 2014 | 1 (ref) | 1.16 (0.97–1.38) | 1.18 (1.00–1.40) | 0.85 (0.68–1.05) | 0.79 (0.68–0.93) | 0.63 (0.50–0.79) | 0.95 (0.71–1.28) |
| 1997-2014 | - | 1.01 (0.97–1.05) | 1.07 (1.04–1.10) | 0.87 (0.85–0.90) | 0.77 (0.75–0.80) | 0.83 (0.80–0.85) | 1.07 (1.02–1.13) |
| Adjusted for sex, age, race/ethnicity, education, marital status, body mass index, smoking status, physical activity, and chronic conditions (heart disease, stroke, cancer, diabetes, hypertension, asthma, emphysema, and chronic bronchitis) | | | | | | | |

| **Table S6.** Hazards ratios for all-cause and cause-specific mortality according to alcohol consumption status among NHIS participants in 1997 to 2014 (excluding participants with heart disease, stroke, cancer, diabetes, hypertension, asthma, emphysema, and chronic bronchitis) | | | | | | | |
| --- | --- | --- | --- | --- | --- | --- | --- |
|  | **Lifetime abstainer** | **Former infrequent drinker** | **Former regular drinker** | **Current infrequent drinker** | **Current light drinker** | **Current moderate drinker** | **Current heavy drinker** |
| All-cause |  |  |  |  |  |  |  |
| Model 1 | 1 (ref) | 0.99 (0.92–1.06) | 1.04 (0.99-1.10) | 0.84 (0.80-0.90) | 0.75 (0.71-0.79) | 0.81 (0.76-0.86) | 1.20 (1.11-1.30) |
| CVD |  |  |  |  |  |  |  |
| Model 1 | 1 (ref) | 0.99 (0.86-1.15) | 1.06 (0.95-1.18) | 0.81 (0.72-0.92) | 0.74 (0.66-0.82) | 0.81 (0.72-0.91) | 1.01 (0.85-1.18) |
| Cancer |  |  |  |  |  |  |  |
| Model 1 | 1 (ref) | 1.03 (0.89-1.19) | 1.07 (0.96-1.21) | 0.93 (0.83-1.04) | 0.83 (0.76-0.92) | 0.88 (0.78-0.98) | 1.24 (1.08-1.43) |
| Chronic lower respiratory tract diseases |  |  |  |  |  |  |  |
| Model 1 | 1 (ref) | 1.00 (0.68–1.48) | 1.08 (0.84–1.40) | 1.00 (0.76–1.32) | 0.60 (0.45–0.79) | 0.67 (0.49–0.92) | 1.04 (0.72–1.49) |
| Accidents/injuries |  |  |  |  |  |  |  |
| Model 1 | 1 (ref) | 1.12 (0.79–1.58) | 0.98 (0.75–1.29) | 0.80 (0.61–1.07) | 0.93 (0.75–1.15) | 0.94 (0.74–1.18) | 1.71 (1.29–2.27) |
| Alzheimer’s disease |  |  |  |  |  |  |  |
| Model 1 | 1 (ref) | 1.08 (0.80–1.48) | 0.86 (0.67–1.10) | 0.90 (0.68–1.18) | 0.66 (0.52–0.85) | 0.69 (0.50–0.95) | 0.57 (0.25–1.32) |
| Diabetes mellitus |  |  |  |  |  |  |  |
| Model 1 | 1 (ref) | 0.96 (0.56–1.65) | 0.75 (0.46–1.22) | 1.07 (0.67–1.70) | 0.53 (0.34–0.83) | 0.66 (0.39–1.11) | 1.03 (0.53–1.99) |
| Influenza and pneumonia |  |  |  |  |  |  |  |
| Model 1 | 1 (ref) | 0.81 (0.49–1.34) | 0.93 (0.65–1.31) | 0.72 (0.48–1.08) | 0.60 (0.43–0.85) | 0.43 (0.28–0.67) | 0.88 (0.52–1.50) |
| Nephritis, nephrotic syndrome, or nephrosis |  |  |  |  |  |  |  |
| Model 1 | 1 (ref) | 0.88 (0.48–1.61) | 0.90 (0.58–1.40) | 0.80 (0.47–1.35) | 0.56 (0.34–0.94) | 0.54 (0.28–1.05) | 0.86 (0.41–1.80) |
| Model 1: Adjusted for sex, age, race/ethnicity, education, marital status, body mass index, smoking status, and physical activity | | | | | | | |

| **Table S7.** Hazards ratios for all-cause and cause-specific mortality according to alcohol consumption status stratified for sex, age, race/ethnicity and smoking status among NHIS participants in 1997 to 2014 | | | | | | | |
| --- | --- | --- | --- | --- | --- | --- | --- |
|  | **Lifetime abstainer** | **Former infrequent drinker** | **Former regular drinker** | **Current infrequent drinker** | **Current light drinker** | **Current moderate drinker** | **Current heavy drinker** |
| **Sex** |  |  |  |  |  |  |  |
| **Men** |  |  |  |  |  |  |  |
| All-cause |  |  |  |  |  |  |  |
| HR (95% CI) | 1 (ref) | 0.98 (0.93-1.04) | 1.10 (1.05-1.15) | 0.92 (0.87-0.97) | 0.81 (0.77-0.85) | 0.85 (0.81-0.89) | 1.17 (1.09-1.25) |
| CVD |  |  |  |  |  |  |  |
| HR (95% CI) | 1 (ref) | 0.94 (0.86-1.02) | 1.06 (0.99-1.14) | 0.90 (0.82-0.98) | 0.78 (0.73-0.84) | 0.77 (0.72-0.84) | 1.00 (0.89-1.12) |
| Cancer |  |  |  |  |  |  |  |
| HR (95% CI) | 1 (ref) | 1.01 (0.90-1.14) | 1.19 (1.08-1.31) | 0.99 (0.89-1.11) | 0.89 (0.81-0.97) | 0.95 (0.87-1.04) | 1.37 (1.20-1.57) |
| Chronic lower respiratory tract diseases |  |  |  |  |  |  |  |
| HR (95% CI) | 1(ref) | 1.05 (0.85–1.30) | 1.03 (0.84–1.26) | 0.95 (0.75–1.21) | 0.74 (0.59–0.91) | 0.85 (0.69–1.06) | 1.00 (0.77–1.30) |
| Accidents/injuries |  |  |  |  |  |  |  |
| HR (95% CI) | 1(ref) | 1.13 (0.86–1.47) | 1.10 (0.88–1.39) | 0.94 (0.72–1.23) | 1.01 (0.81–1.26) | 0.94 (0.75–1.16) | 1.52 (1.14–2.03) |
| Alzheimer’s disease |  |  |  |  |  |  |  |
| HR (95% CI) | 1 (ref) | 1.10 (0.81–1.50) | 1.02 (0.78–1.34) | 0.88 (0.65–1.19) | 0.68 (0.52–0.88) | 0.89 (0.68–1.15) | 0.57 (0.31–1.02) |
| Diabetes mellitus |  |  |  |  |  |  |  |
| HR (95% CI) | 1 (ref) | 1.01 (0.79–1.30) | 1.26 (1.03–1.55) | 1.07 (0.84–1.35) | 0.85 (0.68–1.07) | 0.77 (0.60–0.98) | 1.01 (0.68–1.51) |
| Influenza and pneumonia |  |  |  |  |  |  |  |
| HR (95% CI) | 1 (ref) | 0.91 (0.65–1.27) | 1.17 (0.89–1.54) | 0.84 (0.61–1.16) | 0.73 (0.53–0.99) | 0.66 (0.48–0.89) | 1.10 (0.68–1.79) |
| Nephritis, nephrotic syndrome, or nephrosis |  |  |  |  |  |  |  |
| HR (95% CI) | 1 (ref) | 0.97 (0.70–1.35) | 1.03 (0.80–1.34) | 1.08 (0.78–1.49) | 0.70 (0.52–0.93) | 0.67 (0.48–0.93) | 1.15 (0.75–1.77) |
| **Women** |  |  |  |  |  |  |  |
| All-cause |  |  |  |  |  |  |  |
| HR (95% CI) | 1(ref) | 1.03 (0.99-1.07) | 1.08 (1.04-1.12) | 0.84 (0.81-0.87) | 0.73 (0.71-0.76) | 0.80 (0.75-0.84) | 0.94 (0.87-1.00) |
| CVD |  |  |  |  |  |  |  |
| HR (95% CI) | 1(ref) | 1.02 (0.95-1.10) | 1.12 (1.05-1.19) | 0.86 (0.80-0.92) | 0.73 (0.68-0.78) | 0.78 (0.71-0.87) | 0.85 (0.72-1.00) |
| Cancer |  |  |  |  |  |  |  |
| HR (95% CI) | 1(ref) | 1.08 (0.99-1.18) | 1.10 (1.02-1.19) | 0.91 (0.84-0.98) | 0.82 (0.76-0.89) | 0.88 (0.79-0.99) | 1.06 (0.94-1.21) |
| Chronic lower respiratory tract diseases |  |  |  |  |  |  |  |
| HR (95% CI) | 1(ref) | 0.95 (0.82–1.10) | 1.11 (0.96–1.28) | 0.83 (0.72–0.97) | 0.63 (0.54–0.73) | 0.69 (0.55–0.85) | 0.88 (0.71–1.10) |
| Accidents/injuries |  |  |  |  |  |  |  |
| HR (95% CI) | 1(ref) | 1.04 (0.80–1.35) | 1.04 (0.84–1.30) | 0.77 (0.61–1.00) | 0.91 (0.75–1.11) | 0.90 (0.66–1.21) | 1.43 (1.03–2.00) |
| Alzheimer’s disease |  |  |  |  |  |  |  |
| HR (95% CI) | 1 (ref) | 1.00 (0.83–1.20) | 0.86 (0.72–1.03) | 0.71 (0.60–0.85) | 0.65 (0.55–0.77) | 0.76 (0.58–0.98) | 0.69 (0.46–1.03) |
| Diabetes mellitus |  |  |  |  |  |  |  |
| HR (95% CI) | 1 (ref) | 0.88 (0.72–1.09) | 1.15 (0.94–1.41) | 0.83 (0.66–1.04) | 0.60 (0.47–0.76) | 0.81 (0.55–1.20) | 0.84 (0.43–1.62) |
| Influenza and pneumonia |  |  |  |  |  |  |  |
| HR (95% CI) | 1 (ref) | 0.68 (0.52–0.88) | 0.88 (0.71–1.10) | 0.65 (0.50–0.84) | 0.59 (0.47–0.74) | 0.58 (0.40–0.83) | 0.58 (0.34–1.00) |
| Nephritis, nephrotic syndrome, or nephrosis |  |  |  |  |  |  |  |
| HR (95% CI) | 1 (ref) | 1.03 (0.80–1.31) | 0.92 (0.71–1.18) | 0.96 (0.75–1.24) | 0.64 (0.47–0.87) | 0.36 (0.20–0.65) | 0.71 (0.39–1.28) |
| **Age group** |  |  |  |  |  |  |  |
| **<60 years** |  |  |  |  |  |  |  |
| All-cause |  |  |  |  |  |  |  |
| HR (95% CI) | 1(ref) | 1.03 (0.96-1.11) | 1.12 (1.05-1.18) | 0.85 (0.80-0.90) | 0.73 (0.70-0.77) | 0.80 (0.75-0.85) | 1.12 (1.04-1.20) |
| CVD |  |  |  |  |  |  |  |
| HR (95% CI) | 1(ref) | 0.94 (0.80-1.09) | 1.03 (0.93-1.14) | 0.82 (0.73-0.92) | 0.69 (0.62-0.77) | 0.67 (0.59-0.76) | 0.87 (0.74-1.01) |
| Cancer |  |  |  |  |  |  |  |
| HR (95% CI) | 1(ref) | 1.07 (0.94-1.22) | 1.11 (1.00-1.24) | 0.88 (0.79-0.98) | 0.79 (0.72-0.87) | 0.86 (0.77-0.96) | 1.20 (1.06-1.36) |
| Chronic lower respiratory tract diseases |  |  |  |  |  |  |  |
| HR (95% CI) | 1(ref) | 1.35 (0.98–1.85) | 1.23 (0.95–1.59) | 0.90 (0.70–1.16) | 0.62 (0.47–0.82) | 0.90 (0.66–1.23) | 0.78 (0.55–1.11) |
| Accidents/injuries |  |  |  |  |  |  |  |
| HR (95% CI) | 1(ref) | 1.36 (1.01–1.83) | 1.37 (1.08–1.73) | 0.97 (0.76–1.26) | 1.06 (0.86–1.30) | 1.01 (0.80–1.27) | 1.63 (1.25–2.13) |
| Alzheimer’s disease |  |  |  |  |  |  |  |
| HR (95% CI) | 1 (ref) | 0.89 (0.35–2.25) | 0.60 (0.30–1.20) | 0.67 (0.32–1.42) | 0.75 (0.41–1.36) | 0.87 (0.39–1.94) | 0.14 (0.02–1.05) |
| Diabetes mellitus |  |  |  |  |  |  |  |
| HR (95% CI) | 1 (ref) | 0.99 (0.72–1.35) | 1.25 (1.00–1.58) | 0.97 (0.74–1.26) | 0.70 (0.52–0.93) | 0.85 (0.63–1.16) | 1.20 (0.76–1.87) |
| Influenza and pneumonia |  |  |  |  |  |  |  |
| HR (95% CI) | 1 (ref) | 0.84 (0.48–1.47) | 0.88 (0.57–1.34) | 0.79 (0.50–1.25) | 0.55 (0.35–0.86) | 0.26 (0.15–0.46) | 0.70 (0.35–1.41) |
| Nephritis, nephrotic syndrome, or nephrosis |  |  |  |  |  |  |  |
| HR (95% CI) | 1 (ref) | 0.64 (0.38–1.09) | 0.77 (0.51–1.15) | 0.63 (0.42–0.94) | 0.41 (0.28–0.59) | 0.46 (0.27–0.78) | 0.99 (0.57–1.72) |
| **≥60 years** |  |  |  |  |  |  |  |
| All-cause |  |  |  |  |  |  |  |
| HR (95% CI) | 1(ref) | 1.01 (0.97-1.04) | 1.09 (1.06-1.13) | 0.89 (0.86-0.92) | 0.80 (0.78-0.83) | 0.83 (0.80-0.87) | 1.03 (0.97-1.09) |
| CVD |  |  |  |  |  |  |  |
| HR (95% CI) | 1(ref) | 1.00 (0.94-1.06) | 1.11 (1.05-1.18) | 0.89 (0.84-0.94) | 0.79 (0.75-0.84) | 0.81 (0.76-0.87) | 0.95 (0.86-1.05) |
| Cancer |  |  |  |  |  |  |  |
| HR (95% CI) | 1(ref) | 1.01 (0.92-1.09) | 1.13 (1.05-1.21) | 0.96 (0.89-1.03) | 0.88 (0.82-0.95) | 0.93 (0.86-1.01) | 1.17 (1.05-1.31) |
| Chronic lower respiratory tract diseases |  |  |  |  |  |  |  |
| HR (95% CI) | 1(ref) | 0.91 (0.80–1.05) | 1.01 (0.90–1.14) | 0.88 (0.76–1.02) | 0.71 (0.62–0.81) | 0.75 (0.64–0.88) | 1.01 (0.84–1.21) |
| Accidents/injuries |  |  |  |  |  |  |  |
| HR (95% CI) | 1(ref) | 1.07 (0.84–1.36) | 1.04 (0.85–1.26) | 0.86 (0.68–1.09) | 1.05 (0.84–1.31) | 0.89 (0.70–1.14) | 1.69 (1.18–2.40) |
| Alzheimer’s disease |  |  |  |  |  |  |  |
| HR (95% CI) | 1 (ref) | 1.03 (0.88–1.21) | 0.93 (0.81–1.08) | 0.77 (0.65–0.90) | 0.65 (0.56–0.75) | 0.79 (0.66–0.95) | 0.70 (0.50–1.00) |
| Diabetes mellitus |  |  |  |  |  |  |  |
| HR (95% CI) | 1 (ref) | 0.89 (0.75–1.07) | 1.14 (0.96–1.34) | 0.89 (0.73–1.10) | 0.78 (0.64–0.95) | 0.66 (0.50–0.87) | 0.64 (0.39–1.05) |
| Influenza and pneumonia |  |  |  |  |  |  |  |
| HR (95% CI) | 1 (ref) | 0.74 (0.58–0.94) | 1.03 (0.86–1.24) | 0.67 (0.54–0.83) | 0.67 (0.55–0.81) | 0.70 (0.54–0.91) | 0.89 (0.60–1.31) |
| Nephritis, nephrotic syndrome, or nephrosis |  |  |  |  |  |  |  |
| HR (95% CI) | 1 (ref) | 1.11 (0.90–1.36) | 1.04 (0.85–1.26) | 1.16 (0.92–1.47) | 0.79 (0.63–0.99) | 0.63 (0.47–0.85) | 0.80 (0.52–1.25) |
| **Race/ethnicity** |  |  |  |  |  |  |  |
| **Whites** |  |  |  |  |  |  |  |
| All-cause |  |  |  |  |  |  |  |
| HR (95% CI) | 1(ref) | 0.99 (0.96-1.04) | 1.08 (1.04-1.12) | 0.87 (0.84-0.90) | 0.76 (0.73-0.78) | 0.80 (0.77-0.83) | 1.05 (0.99-1.11) |
| CVD |  |  |  |  |  |  |  |
| HR (95% CI) | 1(ref) | 0.97 (0.91-1.04) | 1.07 (1.01-1.13) | 0.85 (0.80-0.90) | 0.74 (0.69-0.78) | 0.75 (0.70-0.80) | 0.90 (0.82-1.00) |
| Cancer |  |  |  |  |  |  |  |
| HR (95% CI) | 1(ref) | 1.05 (0.97-1.14) | 1.18 (1.09-1.27) | 0.98 (0.91-1.06) | 0.88 (0.82-0.94) | 0.92 (0.85-0.99) | 1.24 (1.13-1.37) |
| Chronic lower respiratory tract diseases |  |  |  |  |  |  |  |
| HR (95% CI) | 1(ref) | 0.99 (0.86–1.13) | 1.07 (0.95–1.21) | 0.89 (0.78–1.03) | 0.68 (0.59–0.78) | 0.78 (0.67–0.91) | 0.99 (0.83–1.17) |
| Accidents/injuries |  |  |  |  |  |  |  |
| HR (95% CI) | 1(ref) | 1.02 (0.83–1.26) | 1.00 (0.84–1.19) | 0.75 (0.62–0.92) | 0.88 (0.74–1.06) | 0.81 (0.67–1.00) | 1.37 (1.09–1.74) |
| Alzheimer’s disease |  |  |  |  |  |  |  |
| HR (95% CI) | 1 (ref) | 1.05 (0.88–1.25) | 0.88 (0.75–1.04) | 0.78 (0.66–0.93) | 0.65 (0.56–0.76) | 0.77 (0.64–0.93) | 0.70 (0.45–1.07) |
| Diabetes mellitus |  |  |  |  |  |  |  |
| HR (95% CI) | 1 (ref) | 0.88 (0.72–1.07) | 1.13 (0.95–1.35) | 0.95 (0.78–1.17) | 0.77 (0.62–0.94) | 0.68 (0.53–0.86) | 0.90 (0.59–1.36) |
| Influenza and pneumonia |  |  |  |  |  |  |  |
| HR (95% CI) | 1 (ref) | 0.78 (0.60–1.01) | 1.02 (0.83–1.25) | 0.71 (0.57–0.89) | 0.61 (0.50–0.75) | 0.58 (0.45–0.76) | 0.81 (0.56–1.17) |
| Nephritis, nephrotic syndrome, or nephrosis |  |  |  |  |  |  |  |
| HR (95% CI) | 1 (ref) | 1.07 (0.85–1.36) | 1.02 (0.82–1.27) | 1.01 (0.78–1.30) | 0.74 (0.57–0.96) | 0.61 (0.45–0.83) | 0.99 (0.66–1.48) |
| **Non-whites** |  |  |  |  |  |  |  |
| All-cause |  |  |  |  |  |  |  |
| HR (95% CI) | 1(ref) | 1.05 (0.99-1.11) | 1.12 (1.06-1.17) | 0.86 (0.81-0.92) | 0.83 (0.78-0.87) | 0.96 (0.89-1.03) | 1.22 (1.11-1.34) |
| CVD |  |  |  |  |  |  |  |
| HR (95% CI) | 1(ref) | 1.02 (0.92-1.13) | 1.15 (1.05-1.26) | 0.95 (0.85-1.07) | 0.85 (0.78-0.94) | 0.92 (0.81-1.06) | 1.14 (0.95-1.37) |
| Cancer |  |  |  |  |  |  |  |
| HR (95% CI) | 1(ref) | 1.11 (0.98-1.25) | 1.14 (1.03-1.26) | 0.86 (0.75-0.99) | 0.82 (0.72-0.92) | 0.98 (0.85-1.12) | 1.28 (1.07-1.53) |
| Chronic lower respiratory tract diseases |  |  |  |  |  |  |  |
| HR (95% CI) | 1(ref) | 1.07 (0.78–1.48) | 1.05 (0.81–1.35) | 0.86 (0.61–1.22) | 0.70 (0.50–0.97) | 0.94 (0.63–1.40) | 0.66 (0.38–1.15) |
| Accidents/injuries |  |  |  |  |  |  |  |
| HR (95% CI) | 1(ref) | 1.14 (0.81–1.61) | 1.17 (0.86–1.60) | 1.17 (0.85–1.62) | 1.14 (0.86–1.51) | 1.19 (0.86–1.65) | 1.66 (1.08–2.56) |
| Alzheimer’s disease |  |  |  |  |  |  |  |
| HR (95% CI) | 1 (ref) | 0.95 (0.67–1.34) | 1.12 (0.84–1.50) | 0.59 (0.40–0.89) | 0.66 (0.44–1.00) | 1.14 (0.65–1.98) | 1.07 (0.49–2.35) |
| Diabetes mellitus |  |  |  |  |  |  |  |
| HR (95% CI) | 1 (ref) | 1.01 (0.80–1.29) | 1.25 (1.01–1.56) | 0.78 (0.59–1.03) | 0.65 (0.49–0.87) | 1.00 (0.71–1.40) | 1.11 (0.65–1.90) |
| Influenza and pneumonia |  |  |  |  |  |  |  |
| HR (95% CI) | 1 (ref) | 0.66 (0.43–1.00) | 0.88 (0.65–1.20) | 0.64 (0.42–0.98) | 0.70 (0.48–1.03) | 0.57 (0.34–0.97) | 0.95 (0.48–1.91) |
| Nephritis, nephrotic syndrome, or nephrosis |  |  |  |  |  |  |  |
| HR (95% CI) | 1 (ref) | 0.84 (0.60–1.17) | 0.91 (0.68–1.21) | 0.97 (0.69–1.37) | 0.45 (0.31–0.65) | 0.53 (0.30–0.96) | 0.92 (0.51–1.69) |
| **Smoking status** |  |  |  |  |  |  |  |
| **Never** |  |  |  |  |  |  |  |
| All-cause |  |  |  |  |  |  |  |
| HR (95% CI) | 1(ref) | 1.00 (0.96-1.05) | 1.06 (1.02-1.11) | 0.84 (0.81-0.88) | 0.75 (0.73-0.78) | 0.77 (0.73-0.81) | 1.06 (0.97-1.16) |
| CVD |  |  |  |  |  |  |  |
| HR (95% CI) | 1(ref) | 1.00 (0.93-1.09) | 1.06 (0.98-1.14) | 0.85 (0.79-0.91) | 0.73 (0.68-0.79) | 0.73 (0.66-0.80) | 0.95 (0.80-1.14) |
| Cancer |  |  |  |  |  |  |  |
| HR (95% CI) | 1(ref) | 1.09 (0.98-1.22) | 1.20 (1.09-1.32) | 0.94 (0.85-1.05) | 0.89 (0.81-0.96) | 0.92 (0.81-1.03) | 1.24 (1.02-1.52) |
| Chronic lower respiratory tract diseases |  |  |  |  |  |  |  |
| HR (95% CI) | 1(ref) | 0.91 (0.69–1.20) | 0.97 (0.76–1.24) | 0.80 (0.61–1.07) | 0.71 (0.53–0.95) | 0.50 (0.32–0.78) | 0.50 (0.23–1.09) |
| Accidents/injuries |  |  |  |  |  |  |  |
| HR (95% CI) | 1(ref) | 1.03 (0.80–1.34) | 1.22 (0.96–1.55) | 0.87 (0.68–1.10) | 0.97 (0.79–1.20) | 0.85 (0.64–1.13) | 1.45 (1.01–2.09) |
| Alzheimer’s disease |  |  |  |  |  |  |  |
| HR (95% CI) | 1 (ref) | 0.96 (0.79–1.17) | 0.88 (0.72–1.08) | 0.75 (0.62–0.90) | 0.64 (0.53–0.77) | 0.80 (0.62–1.04) | 0.61 (0.36–1.06) |
| Diabetes mellitus |  |  |  |  |  |  |  |
| HR (95% CI) | 1 (ref) | 0.91 (0.74–1.13) | 1.01 (0.81–1.25) | 0.93 (0.73–1.19) | 0.70 (0.56–0.88) | 0.75 (0.53–1.06) | 1.13 (0.62–2.05) |
| Influenza and pneumonia |  |  |  |  |  |  |  |
| HR (95% CI) | 1 (ref) | 0.74 (0.54–1.00) | 0.96 (0.76–1.22) | 0.59 (0.44–0.78) | 0.51 (0.39–0.68) | 0.59 (0.39–0.88) | 0.77 (0.36–1.68) |
| Nephritis, nephrotic syndrome, or nephrosis |  |  |  |  |  |  |  |
| HR (95% CI) | 1 (ref) | 0.81 (0.62–1.07) | 0.93 (0.72–1.19) | 0.98 (0.74–1.30) | 0.65 (0.49–0.87) | 0.50 (0.31–0.79) | 0.70 (0.32–1.54) |
| **Ever smoked** |  |  |  |  |  |  |  |
| All-cause |  |  |  |  |  |  |  |
| HR (95% CI) | 1(ref) | 1.02 (0.97-1.07) | 1.08 (1.04-1.13) | 0.91 (0.87-0.96) | 0.78 (0.75-0.82) | 0.86 (0.82-0.90) | 1.05 (1.09-1.22) |
| CVD |  |  |  |  |  |  |  |
| HR (95% CI) | 1(ref) | 0.98 (0.91-1.06) | 1.09 (1.02-1.17) | 0.91 (0.84-0.99) | 0.79 (0.73-0.85) | 0.82 (0.76-0.88) | 1.00 (0.90-1.11) |
| Cancer |  |  |  |  |  |  |  |
| HR (95% CI) | 1(ref) | 0.99 (0.91-1.09) | 1.06 (0.98-1.15) | 0.93 (0.86-1.02) | 0.81 (0.74-0.88) | 0.89 (0.82-0.96) | 1.28 (1.15-1.42) |
| Chronic lower respiratory tract diseases |  |  |  |  |  |  |  |
| HR (95% CI) | 1(ref) | 1.03 (0.89–1.19) | 1.05 (0.92–1.20) | 0.92 (0.79–1.07) | 0.67 (0.58–0.77) | 0.83 (0.70–0.97) | 1.07 (0.90–1.27) |
| Accidents/injuries |  |  |  |  |  |  |  |
| HR (95% CI) | 1(ref) | 1.09 (0.83–1.43) | 0.97 (0.78–1.21) | 0.82 (0.63–1.08) | 0.92 (0.73–1.15) | 0.90 (0.71–1.14) | 1.49 (1.15–1.94) |
| Alzheimer’s disease |  |  |  |  |  |  |  |
| HR (95% CI) | 1 (ref) | 1.11 (0.85–1.46) | 0.96 (0.77–1.21) | 0.79 (0.60–1.03) | 0.68 (0.53–0.87) | 0.83 (0.64–1.08) | 0.69 (0.45–1.06) |
| Diabetes mellitus |  |  |  |  |  |  |  |
| HR (95% CI) | 1 (ref) | 0.96 (0.75–1.24) | 1.26 (1.03–1.55) | 0.94 (0.73–1.20) | 0.78 (0.61–0.98) | 0.78 (0.59–1.04) | 0.98 (0.65–1.47) |
| Influenza and pneumonia |  |  |  |  |  |  |  |
| HR (95% CI) | 1 (ref) | 0.82 (0.59–1.13) | 1.07 (0.82–1.39) | 0.87 (0.64–1.17) | 0.77 (0.57–1.03) | 0.64 (0.46–0.88) | 0.97 (0.65–1.44) |
| Nephritis, nephrotic syndrome, or nephrosis |  |  |  |  |  |  |  |
| HR (95% CI) | 1 (ref) | 1.24 (0.92–1.66) | 1.06 (0.79–1.41) | 1.11 (0.82–1.51) | 0.72 (0.51–1.02) | 0.69 (0.47–1.00) | 1.19 (0.76–1.84) |
| Adjusted for sex, age, race/ethnicity, education, marital status, body mass index, physical activity, smoking, and physician-diagnosed diseases (heart disease, stroke, cancer, diabetes, hypertension, asthma, emphysema, and chronic bronchitis) | | | | | | | |
